# Conserved active site cysteine residue of archaeal THI4 homolog is essential for thiamine biosynthesis in *Haloferax volcanii*

**DOI:** 10.1186/s12866-014-0260-0

**Published:** 2014-10-28

**Authors:** Sungmin Hwang, Bryan Cordova, Nikita Chavarria, Dina Elbanna, Stephen McHugh, Jenny Rojas, Friedhelm Pfeiffer, Julie A Maupin-Furlow

**Affiliations:** Department of Microbiology and Cell Science, University of Florida, Gainesville, FL 32611-0700 USA; Genetics Institute, University of Florida, Gainesville, FL 32611-0700 USA; Department of Membrane Biochemistry, Max Planck Institute of Biochemistry, Am Klopferspitz 18, D-82152 Martinsried, Germany

**Keywords:** Vitamin B1, Coenzyme biosynthesis, Thiamine, Sulfur relay, Archaea

## Abstract

**Background:**

Thiamine (vitamin B1) is synthesized *de novo* by certain yeast, fungi, plants, protozoans, bacteria and archaea. The pathway of thiamine biosynthesis by archaea is poorly understood, particularly the route of sulfur relay to form the thiazole ring. Archaea harbor structural homologs of both the bacterial (ThiS-ThiF) and eukaryotic (THI4) proteins that mobilize sulfur to thiazole ring precursors by distinct mechanisms.

**Results:**

Based on comparative genome analysis, halophilic archaea are predicted to synthesize the pyrimidine moiety of thiamine by the bacterial pathway, initially suggesting that also a bacterial ThiS-ThiF type mechanism for synthesis of the thiazole ring is used in which the sulfur carrier ThiS is first activated by ThiF-catalyzed adenylation. The only ThiF homolog of *Haloferax volcanii* (UbaA) was deleted but this had no effect on growth in the absence of thiamine. Usage of the eukaryotic THI4-type sulfur relay was initially considered less likely for thiamine biosynthesis in archaea, since the active-site cysteine residue of yeast THI4p that donates the sulfur to the thiazole ring by a suicide mechanism is replaced by a histidine residue in many archaeal THI4 homologs and these are described as D-ribose-1,5-bisphosphate isomerases. The THI4 homolog of the halophilic archaea, including *Hfx. volcanii* (HVO_0665, HvThi4) was found to differ from that of methanogens and thermococci by having a cysteine residue (Cys165) corresponding to the conserved active site cysteine of yeast THI4p (Cys205). Deletion of HVO_0665 generated a thiamine auxotroph that was *trans*-complemented by a wild-type copy of HVO_0665, but not the modified gene encoding an HvThi4 C165A variant.

**Conclusions:**

Based on our results, we conclude that the archaeon *Hfx. volcanii* uses a yeast THI4-type mechanism for sulfur relay to form the thiazole ring of thiamine. We extend this finding to a relatively large group of archaea, including haloarchaea, ammonium oxidizing archaea, and some methanogen and *Pyrococcus* species, by observing that these organisms code for THI4 homologs that have a conserved active site cysteine residue which is likely used in thiamine biosynthesis. Thus, archaeal members of IPR002922 THI4 family that have a conserved cysteine active site should be reexamined for a function in thiamine biosynthesis.

**Electronic supplementary material:**

The online version of this article (doi:10.1186/s12866-014-0260-0) contains supplementary material, which is available to authorized users.

## Background

Thiamine pyrophosphate (TPP) is a cofactor found in virtually all living systems and is important to the function of enzymes in glycolysis, the citric acid cycle (TCA), amino acid biosynthesis, and other metabolic pathways [1]. Many prokaryotes and some eukaryotes (plants and fungi) can synthesize TPP [2], whereas mammals and other vertebrates require this cofactor in their diet in the form of vitamin B1 (thiamine) [3]. This “thio-“or “sulfur-containing” vitamin consists of a thiazole ring linked to a pyrimidine, with the moieties synthesized separately and then coupled to form thiamine [2].

Thiazole biosynthesis in bacteria is well characterized and involves a complex chain of oxidative condensation reactions that use 1-deoxy-D-xylulose-5-phosphate, glycine (or tyrosine) and a sulfur source [2]. Sulfur is thought to be derived from cysteine and incorporated into the thiazole ring by a series of enzyme-mediated sulfur transfer steps that include ThiS, ThiF, ThiG, ThiI, and IscS (NifS). In this process, ThiS is adenylated at its C-terminus by ThiF in a mechanism analogous to the activation of ubiquitin. The adenylated ThiS is converted to a thiocarboxylate at the C-terminal glycine residue by sulfur transfer from ThiI and cysteine desulfurase (*e.g*., IscS, NifS). ThiS thiocarboxylate serves as a sulfur donor for formation of the thiazole ring via thiazole synthase (ThiG).

Molecular details of thiazole ring formation in fungi, yeast and plants are revealed by study of THI4 family proteins. Early work demonstrated that THI4 gene expression is repressed by thiamine and that disruption of this gene in yeast generates strains that are auxotrophic for thiamine, but able to grow in the presence of a thiazole precursor of thiamine [4]. Thus, yeast THI4p was examined for thiazole ring formation and found to catalyze a single turnover reaction in which THI4p Cys205 serves as the source of sulfur to form a thiazole precursor of thiamine with NAD and glycine as co-substrates [5].

Thiazole biosynthesis in archaea is poorly understood. Most archaea have homologs that cluster to the THI4 family (IPR002922) of proteins, but lack the conserved cysteine residue of the yeast THI4p that is required for sulfur transfer in formation of the thiazole ring and instead have a histidine residue which is well conserved. There are conflicting reports on the function of these archaeal THI4 homologs. Initially, the proteins from *Methanocaldococcus jannaschii* (MJ0601) and *Methanosarcina acetivorans* (MA_2851) were reported to convert ribose-1,5-bisphosphate (R15P) into ribulose-1,5-bisphosphate, the substrate of ribulose-1,5-bisphosphate carboxylase/oxygenase (RuBisCO) [6]. With respect to the experimental details, however, the authors did not measure the assumed activity of a R15P isomerase (which might also be called ribulose-1,5-bisphosphate synthase) but measured stimulation of incorporation of labeled CO_2_ into an acid-stable compound which was identified as PGA (3-phosphoglyceric acid), the product of RuBisCO. As an additional complication, the starting product 5-phosphoryl-1-pyrophosphate had to be subjected to heat treatment prior to performing the assay. Despite this ambiguity, the conclusions of the initial report with respect to the existence of an archaeal R15P isomerase activity have been confirmed in a subsequent study [7]. These authors directly measured R15P isomerase activity and even put this activity into the wider context of an AMP metabolic pathway (together with RuBisCO and the *deoA* gene product). However, they assigned the R15P isomerase activity to a completely different protein. This protein, referred to as TK-e2b2, is TK0185 (as retrieved from NCBI using BAD84374, the code provided for this protein) [7]. TK0185 clusters with the e2b2 family (IPR005250) and has no resemblance whatsoever with members of the THI4 family. TK0185 was active in an *in vitro* R15P isomerase assay. The authors also tested TK0434, the *Thermococcus* THI4 family member and ortholog of MJ0601, in their *in vitro* R15P isomerase assay, but did not detect any activity. In addition, they point out that the three enzymes of their AMP metabolic pathway (RuBisCO, e2b2 (TK0185) and DeoA) share a common phylogenetic profile while TK0434 does not. This implies that the original function assignment to MJ0601 and MA_2851 as R15P isomerase may not be correct. Whatever the function of MJ0601 and the other THI4 members with a histidine rather than cysteine may be, there is not the slightest hint that it may be related to thiamine biosynthesis.

Here we report that archaeal THI4 homologs with a conserved catalytic cysteine residue are linked to thiamine biosynthesis. Deletion of the HvThi4 gene homolog (HVO_0665) of *Haloferax volcanii* was found to confer an auxotrophic requirement for thiamine. The conserved active site cysteine residue of HvThi4 (Cys165) was needed to restore the growth of this thiamine autotroph to wild-type levels. Overall, our results provide new insight that archaeal THI4 family (IPR002922) members with conserved active site cysteine residues are important in thiamine biosynthesis and that most halophilic archaea and ammonia oxidizing archaea (AOA) are likely to use a THI4-type mechanism for sulfur relay to the thiazole ring.

## Methods

### Materials

Biochemicals were from Sigma-Aldrich (St. Louis, MO). Other organic and inorganic analytical grade chemicals were from Fisher Scientific (Atlanta, GA). Restriction endonucleases, T4 DNA ligase and Phusion polymerase were from New England Biolabs (Ipswich, MA). Taq DNA polymerase was from Bioline (Taunton, MA). Pfu DNA polymerase was from Stratagene (La Jolla, CA). Desalted oligonucleotides were from Integrated DNA Technologies (Coralville, IA). Agarose for routine analysis of DNA was from Bio-Rad Laboratories (Hercules, CA).

### Strains and media

Strains used in this study are summarized in Table 1. *E. coli* GM2163 was used for replication of plasmid DNA prior to transformation into *Hfx. volcanii* according to standard methods [8]. *E. coli* strains were grown at 37°C in Luria-Bertani (LB) medium supplemented with ampicillin (Amp, 0.1 mg · ml^−1^) as needed. *Hfx. volcanii* strains were grown at 42°C in ATCC 974 complex medium supplemented with novobiocin (Nv, 0.2 μg · ml^−1^), ammonium chloride (5 mM)-containing glycerol minimal medium supplemented with and without thiamine (0.8 μg∙ml^−1^) (GMM ± thiamine), and CA medium as previously described [9]. Medium formulae were according to *The Halohandbook* [8] with the following modification: 20 mM glycerol served as the sole carbon source. Uracil dissolved in 100% (v/v) DMSO to 50 mg∙ml^−1^ was supplemented in growth media to a final concentration of 50 μg∙ml^−1^ as needed. Cells were grown in liquid cultures with rotary shaking at 200 rpm and on solid medium (1.5% [w/v] agar plates). To measure thiamine-dependent growth, strains were grown in complex medium to log-phase (4 ml ATCC 974 in 13 × 100 mm tubes), washed twice with GMM without thiamine by centrifugation (8,600 × *g*, 2 min at room temperature), subcultured to a starting OD_600_ of 0.03 in 25 ml of GMM ± thiamine in a 250-ml Erlenmeyer baffled flask, and incubated at 42°C. Once the cells reached log-phase, the strains were re-inoculated into fresh medium (GMM ± thiamine) for further analysis of growth (in 250-ml Erlenmeyer baffled flasks with incubation at 42°C). Growth was monitored by measuring OD_600_ over time (where 1 OD_600_ unit equals approximately 1 × 10^9^ colony forming units (CFU) · ml^−1^) and generation times were calculated. All experiments were performed in triplicate and the mean ± standard deviation (S.D.) was calculated.

**Table 1:** **Strains and plasmids used in this study** ^**a**^

**Strain, plasmid or primer**	**Description**	**Source or reference**
***E. coli***		
TOP10	F^−^ *recA1 endA1 hsdR17*(r_K_^−^ m_K_^+^) *supE44 thi-1 gyrA relA1*	Invitrogen
GM2163	F^−^ *ara-14 leuB6 fhuA31 lacY1 tsx78 glnV44 galK2 galT22 mcrA dcm-6 hisG4 rfbD1 rpsL136 dam13*::Tn*9 xylA5 mtl-1 thi-1 mcrB1 hsdR2*	New England Biolabs
XL1-Blue	*endA1 gyrA96*(*nal* ^R^) *thi-1 recA1 relA1 lac glnV44* F'[::Tn10 *proAB* ^+^ *lacI* ^q^ Δ(*lacZ*)M15] *hsdR17*(r_K_^−^ m_K_^+^)	Stratagene
***Hfx. volcanii***		
DS70	wild-type isolate DS2 cured of plasmid pHV2	[10]
H26	DS70 *ΔpyrE2*	[9]
NC1011	H26 *Δthi4*	This study
HM1052	H26 *ΔubaA*	[11]
**Plasmids**		
pTA131	Ap^r^; pBluescript II carries P_*fdx*_-*pyrE2* with MCS	[9]
pJAM202c	Ap^r^; Nv^r^; *Hfx. volcanii*-*E. coli* shuttle plasmid, empty vector control	[12]
pJAM809	Ap^r^; Nv^r^; pJAM202c carries P2_*rrnA*_-*hvo_1862*-*strepII*	[13]
pJAM2819	Ap^r^; pTA131-based plasmid for pre-deletion of *hvo_0665*	This study
pJAM2820	Ap^r^; pTA131-based plasmid for *Δhvo_0665*	This study
pJAM2821	Ap^r^; Nv^r^; pJAM809-derived carries P2_*rrnA*_-*hvo_0665-strepII* (which replaced *hvo_1862-strepII*)	This study
pJAM2822	Ap^r^; Nv^r^; pJAM2821-derived carries P2_*rrnA*_-*hvo_0665 C165A-strepII*	This study

^a^Abbreviations: Ap^r^, ampicillin resistance; Nv^r^, novobiocin resistance; −*strepII*, C-terminal StrepII-tag fusion coding sequence (preceded by a KpnI site that facilitated generation of the C-terminal StrepII fusion with GlyThr linker). HvThi4 corresponds to HVO_0665, the gene locus tag number of GI: 292654829.

### DNA-based methods

Plasmids and primers used in this study are summarized in Tables 1 and 2. Plasmid DNA was isolated by QIAprep Spin Miniprep kit according to manufacturer’s protocols (Qiagen, Valencia, CA). Polymerase chain reaction (PCR) with an iCycler (BioRad Laboratories) was according to standard methods using template DNA and primer pairs as indicated in Table 2. Genomic DNA was extracted from *Hfx. volcanii* strains by boiling colonies resuspended in ddH_2_O or by DNA spooling, as described in *The Halohandbook* [8]. Phusion and Pfu DNA polymerases were used for high-fidelity PCR-based cloning, and Taq DNA polymerase was used for colony screening. DNA fragments were compared to Hi-Lo DNA molecular weight markers (Minnesota Molecular, Minneapolis, MN) by electrophoresis (90 V, 30–45 min) using 0.8-2% (w/v) agarose gels in TAE buffer [40 mM Tris, 20 mM acetic acid, and 1 mM ethylenediaminetetraacetic acid (EDTA), pH 8.0]. Gels were stained with ethidium bromide at 0.25 μg∙ml^−1^ and visualized with a Mini visionary imaging system (FOTODYNE, Hartland, WI). DNA fragments were isolated by MinElute PCR purification (Qiagen) or from 0.8% (w/v) SeaKem GTG agarose (FMC Bioproducts, Rockland, ME) gels in TAE buffer at pH 8.0 using the QIAquick gel extraction kit (Qiagen) as needed. The fidelity of all DNA plasmid constructs was verified by Sanger DNA Sequencing (Eton Bioscience, Inc. and UF ICBR DNA sequencing core).

**Table 2:** **Primer pairs used in this study** ^**a**^

**Primer pair**	**PCR Product/Description**	**Primer sequences**
P1: HVO_0665 preKO BamHI RV1	Primers anneal ~500 bp upstream and downstream of *hvo_0665*; used with H26 genomic DNA as template to generate pJAM2819 (pre-deletion) by ligation into HindIII to BamHI sites of pTA131	5’- ATG*AAGCTT*AACGCGAGTCTCCTGTGGGCGCTCGG-3'
P2: HVO_0665 HindIII preKO FW1		5’-ATT*GGATCC*GACGCGCGCACCTCGCCGTTC-3’
P4: HVO_0665 3’-inverse	Used to generate plasmid pJAM2820 (*Δhvo_0665*) by inverse PCR using pJAM2819 as a template	5’-TCCCGCGCCGGCCGACGACTGA-3’
P3: HVO_0665 5’-inverse		5’-TCCGTCGCGTCGGTGAAGCCGTCGAACGACAT-3’
P7: HVO_0665 700 bp confirm FW	Anneal ~700 bp upstream and downstream of *hvo_0665*; used to confirm *Δhvo_0665*	5’-GCTCGGCGGGGCGAACACG-3
P8: HVO_0665 700 bp confirm RV		5’-GTGACCCACGAGACGACCCACGCG-3’
P5: HVO_0665 NdeI	Used to screen for *Δhvo_0665* and generate pJAM2821 (*in trans* complement of *Δhvo_0665*)	5’-GGGCGGCATATGTCGTTCGACGGCTTCAC-3’
P6: HVO_0665 KpnI		5’-TTGGTACCGTCGTCGGCCGGCGC-3’
P7: C165A THI4 FW	Used to generate pJAM2822 for synthesis of the site-directed variant HvThi4 C165A	5’- CGCGAACTCACG*GCG*GTCGACCCCATC-3’
P8: C165A THI4 RV		5’- GATGGGGTCGAC*CGC*CGTGAGTTCGCG-3’

^a^Italic in the sequences of primers P1/P2 and P7/P8 indicates the introduced BamHI and HindIII sites for ligation into pTA131 and mutation resulting to Cys to Ala conversion, respectively.

### Generation of an *Hfx. volcanii* Thi4 deletion

For generation of an *Hfx. volcanii Δthi4* (*Δhvo_0665*) strain, a *pyrE2*-based pop-in/pop-out deletion strategy was used [9]. In brief, plasmid pJAM2819 was constructed by ligation of a 921-bp DNA fragment that was specific for *thi4* and its 5’ and 3’ flanking regions (500 bp each) into the BamHI to HindIII sites of the *pyrE2*-based plasmid pTA131. The resulting pre-deletion plasmid (pJAM2819) was subsequently used as a template to construct the deletion plasmid pJAM2820, which has the *thi4* gene deleted by inverse PCR. Plasmid pJAM2820 was used for *pyrE2*-based recombination and deletion of *thi4* on the chromosome of *Hfx. volcanii* H26. Strain fidelity was confirmed by PCR with primer pairs that annealed within the *thi4* ORF (P5/P6) and 700 bp upstream and downstream of *thi4* (P7/P8) (Table 2).

### Generation of *Hfx. volcanii* THI4 homolog expression plasmids

The *thi4* gene was isolated from *Hfx. volcanii* genomic DNA by PCR with primers P5/P6 (Table 2). The PCR product was ligated into the NdeI to KpnI sites of plasmid pJAM809, which carries coding sequence for a C-terminal StrepII tag that includes a GT linker (−GTWSHPQPEK, −StrepII) and a ribosomal RNA P2 promoter to drive transcription. The resulting plasmid pJAM2811 was used for *trans*-expression of HvThi4-StrepII in *Hfx. volcanii*. For site-directed mutagenesis, plasmid pJAM2811 was isolated from *E. coli* TOP10 and used as template for PCR with primer pair P7/P8 as indicated in Table 2. Pfu DNA polymerase was used to generate amplicons with the desired single-point mutations as described in the QuikChange Lightning mutagenesis protocol (Stratagene, La Jolla, CA). PCR products were incubated with DpnI at 37°C for 2 h, purified using QIAquick purification kit (Qiagen), and transformed into *E. coli* XL1-Blue. Plasmid pJAM2812 encoding HvThi4 C165A-StrepII was verified by DNA sequencing.

### Comparative genomics

UniProtKB/Swiss-Prot accession numbers associated with protein sequences analyzed in this study are listed in Additional file 1: Tables S1 and S2. Representative archaeal protein sequences that cluster to known enzymes of thiamine biosynthesis and salvage pathways were retrieved from InterPro [14] and from GenBank by Basic Local Alignment Search Tool using BlastP (protein-protein BLAST) [15]. Protein sequences were aligned using ClustalW [16]. Prior to dendrogram construction, extensive gaps and N- and C-terminal extensions were removed using BioEdit [17]. A neighborhood-joining tree that best fit the distance data was constructed by pairwise comparisons using MEGA 5 [18]. The mean genetic distance was calculated using p-distance, and gaps were analyzed by pairwise deletion.

### Fold recognition and 3D-structural modeling

Phyre2 (Protein Homology/AnalogY Recognition Engine V 2.0) web-based server [19,20] was used for fold recognition and model building of HvThi4 (HVO_0665) as well as its homologs, TK0434 and MA_2851. In brief, the primary amino acid sequences were submitted to the Phyre2 server using intensive mode. The Phyre2 threading server combined HHsearch for remote homology detection based on pairwise comparison of hidden Markov models (HMM) with *ab initio* and multiple-template modeling. The library of known protein structures for comparison was from the Protein Data Bank (PDB) and Structural Classification of Proteins (SCOP) databases. Chimera 1.7 [21] was used as an interface for interactive visualization and analysis of molecular 3D protein structures. Conserved active site residues were identified based on biochemical and structural analysis of ScTHI4p (PDB: 3FPZ) and *Neurospora crassa* (PDB: 3JSK, also named CyPBP37).

### SDS-PAGE and Western blotting analysis

*Hfx. volcanii* strains were grown in complex medium to stationary-phase (OD_600_ of 3.0-3.5 in 4 ml ATCC 974 in 13 × 100 mm tubes), harvested by centrifugation (14,500 × *g*, 2 min at room temperature). Cell pellets were boiled in reducing SDS buffer (2% w/v SDS, 10% v/v glycerol, 5% v/v β-mercaptoethanol, 0.002% w/v bromphenol blue and 62.5 mM Tris–HCl, pH 6.8). Proteins were separated by 12% SDS-PAGE and transferred to Hybond-P polyvinylidene fluoride (PVDF) membranes (GE Healthcare Bio-Sciences, Piscataway, NJ) at 4°C for 2.5 h at 90 V by tank blot. Ponceau Red S Stain was used to confirm equal transfer (Boston Bioproducts, Ashland, MA). StrepII-tagged proteins were detected using unconjungated rabbit anti-StrepII polyclonal antibody (Genscript USA, Piscataway, NJ) and alkaline phosphatase (AP)-linked goat anti-rabbit IgG (H + L) antibody (SouthernBiotech, Birmingham, AL), as primary and secondary antibodies respectively. AP signals were detected by chemiluminescence with CDP-Star according to supplier’s protocol (Applied Biosystems, Carlsbad, CA) and visualized with X-ray film (Hyperfilm, GE Healthcare Bio-Sciences). Equivalent protein loading was further confirmed by staining samples similarly separated by SDS-PAGE in gel with Coomassie Blue R-250.

## Results and discussion

### Two distinct mechanisms for incorporating sulfur into the thiazole ring of thiamine predicted in select archaea by genomic reconstruction

An updated genomic comparison of thiamine metabolism homologs of select archaea was performed (Additional file 1). Based on this analysis, *Hfx. volcanii* and related archaea were predicted to use bacterial related enzymes for synthesis of the pyrimidine moiety (HMP-PP, 4-amino-hydroxymethyl-2-methylpyrimidine pyrophosphate) of thiamine. By contrast, the mechanism used for formation of 4-methyl-5-(β-hydroxyethyl) thiazole phosphate (HET-P or THZ-P) containing the thiazole ring was less clear for these organisms. A critical step in formation of the thiazole ring is the incorporation of sulfur. Genomic reconstruction detected two distinct mechanisms for sulfur transfer to synthesize the thiazole ring including: a bacterial ThiF-ThiS related system (UbaA-SAMP) and a yeast THI4p-type mechanism (HvThi4, HVO_0665). However, a conundrum existed in that the UbaA-SAMP system lacked a predicted interaction partner (such as the bacterial ThiG) and that archaeal THI4 homologs (such as MJ0601 and MA_2851) are reported to function as R15P isomerases [6], thus being unrelated to thiamine biosynthesis. To shed some light into this enigma, we analyzed the members of the Thi4 protein family in more detail.

### Haloarchaea and other select archaea have THI4 homologs with a conserved active site cysteine

**Figure 1:**
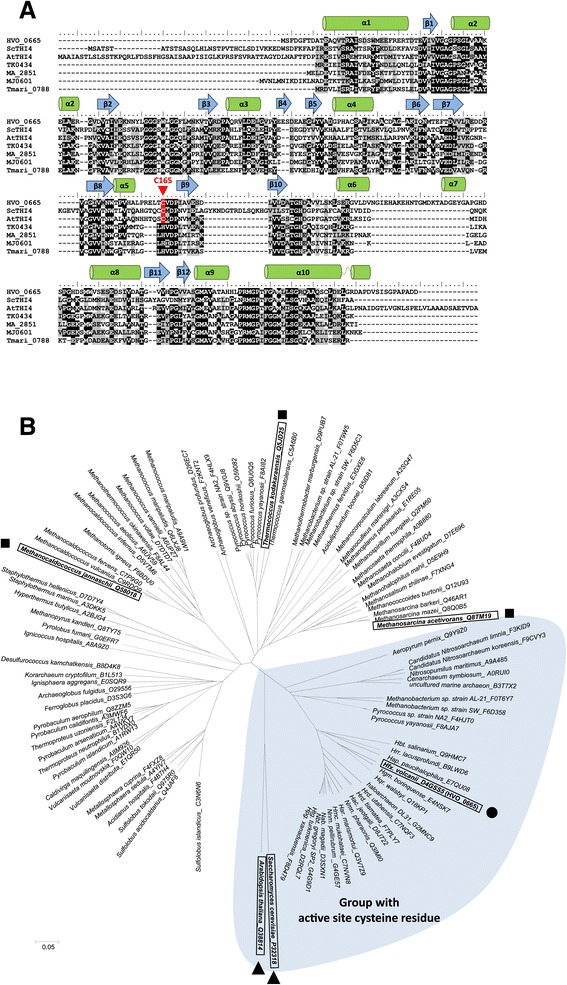
***Haloferax volcanii*** **HVO_0665 (HvThi4) is related to members of the THI4 protein family (IPR002922). (A)** Multiple amino acid sequence alignment of THI4 homologs including *Hfx. volcanii* HVO_0665 (HvThi4), *Saccharomyces cerevisiae* ScTHI4, *Arabidopsis thaliana* AtTHI4, *Thermotoga maritima* Tmari_0788, *Methanosarcina acetivorans* MA_2851, *Methanocaldococcus jannaschii* MJ0601 and *Thermococcus kodakarensis* TK0434. Identical and functionally similar amino acid residues are highlighted in black and grey, respectively, with residues conserved with the ScTHI4 Cys205 active site highlighted in red. α helices and β sheets predicted for HVO_0665 by Phyre2-based homology modeling are indicated above the alignment. **(B)** Cluster analysis of HvThi4 with members of the THI4 protein family. HVO_0665 (HvThi4) of this study is indicated by a circle (●). *M. acetivorans* MA_2851 and *M. jannaschii* MJ0601 described as D-ribose-1,5-bisphosphate isomerases and the associated *T. kodakarensis* TK0434 demonstrated to lack this activity are indicated by squares (■). *S. cerevisiae* and *A. thaliana* THI4 enzymes of thiamine biosynthesis are indicated by triangles (▲). Cluster of archaeal THI4 homologs with a conserved active site cysteine residue analogous to ScTHI4 Cys205 are shaded in blue and include uncharacterized proteins of halophilic archaea, Thaumarchaeota, Aeropyrum, and select methanogens and pyrococci. Three letter genus abbreviations are used as proposed by the Subcommittee on the taxonomy of the family Halobacteriaceae. N- and C-termini were trimmed for protein alignments. UniProtKB accession numbers associated with protein sequences are listed in supplemental information.

To determine the metabolic potential of the archaeal members of the THI4 protein family (IPR002922) in thiamine biosynthesis, archaeal homologs were compared to THI4p of yeast (*Saccharomyces cerevisiae*, ScTHI4) and plant (*Arabidopsis thaliana*, AtTHI4) by multiple amino acid sequence alignment and cluster analysis (Figure 1A-B). By these comparisons, the majority of archaeal THI4 homologs were found to have a histidine residue conserved in the position of the active site cysteine residue (Cys205) of the yeast THI4p, with some even having a proline residue. Archaeal Thi4 ‘histidine-containing’ homologs from methanogenic archaea (MJ0601 and MA_2851) are implicated in R15P isomerase reactions not associated with thiamine metabolism to provide ribulose-bisphosphate as a substrate of archaeal ribulose-bisphosphate carboxylase [6]. In a subsequent study, archaeal R15P isomerases were put into a wider context of CO_2_ fixation via the AMP pathway [7]. Phylogenetic profile analysis as well as *in vivo* and *in vitro* studies assigned R15P isomerase activity to TK0185, a protein unrelated to THI4p [7,22]. By contrast, the THI4p homolog of *T. kododaraensis* (TkThi4, TK0434) did not show any R15P isomerase activity by *in vitro* analysis [7]. Even though the results of these earlier studies are controversial [6,7], none implicates a THI4p homolog to be involved in thiamine biosynthesis in an archaeon.

The ScTHI4p Cys205 residue is highly conserved among eukaryotic THI4 homologs, is required for THI4 activity, and is thought to be used as a sulfur donor in an iron-mediated sulfur transfer reaction to form the thiazole ring, based on MS-based detection of a dehydroalanine residue at this position after a single-turnover reaction [5]. Since the majority of archaeal THI4 homologs lack the conserved active site cysteine, these enzymes are not predicted to use a yeast THI4p-type mechanism of thiazole biosynthesis. However, a subset of archaeal THI4 homologs was detected that displayed not only an overall amino acid sequence similarity to ScTHI4, but also harbored a cysteine residue analogous to the ScTHI4 active site Cys205. For example, the *Hfx. volcanii* THI4 homolog (HvThi4, HVO_0665) had a conserved active site cysteine residue (Cys165) and 31% overall amino acid sequence identity to ScTHI4p with a BLAST query coverage of 92% and E value of 3e-26. The subset of archaeal THI4 homologs with the conserved active site cysteine residue was found to cluster (Figure 1B) and included THI4 homologs of all of the haloarchaea and ammonium-oxidizing Thaumarchaeota examined as well as the crenarchaeote *Aeropyrum pernix*, and select euryarchaeota including *Pyrococcus* sp. strain NA2 and *P. yayanosii* strain CH1 as well as *Methanobacterium* sp. strains AL-21 and SWAN-1 (Figure 1B). These select species of *Pyrococcus* and *Methanobacterium* had two THI4 homologs (one with a conserved active site cysteine residue, clustering with the haloarchaeal homologs, and the other with a conserved histidine residue, clustering with the other His-containing family members (Figure 1B).

**Figure 2:**
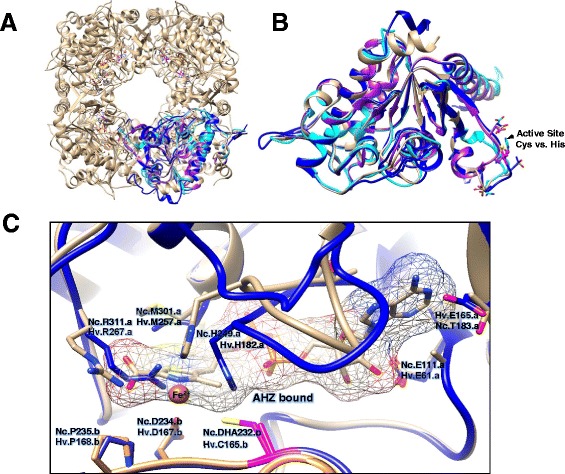
**3D-structural models of archaeal THI4 family proteins compared to the X-ray structure of** ***Neurospora crassa*** **(Nc) THI4p (PDB: 3JSK).** Proteins are represented in ribbon diagram including HVO_0665 (HvThi4, dark blue), MA_2851 (cyan), TK0434 (purple) and NcTHI4p (light brown), with the latter in octameric **(A)** and monomeric **(B)** configuration. For clarity in panel B, N- and C-terminal amino acid extensions of HvThi4 (residues 1–9 and 298–307) and NcTHI4p (residues 35–57) are hidden. **(C)** NcTHI4p residues bound or in close proximity to adenosine diphosphate 5-(beta-ethyl)-4-methyl-thiazole-2-carboxylic acid (AHZ) are indicated with structurally analogous residues of HvThi4 highlighted (where .a and .b indicate residues of chains a and b at the dimer interface). The conserved catalytic cysteine residue of NcTHI4p (Cys232) that is essential for thiamine biosynthesis is in the sulfur minus 2,3-didehydroalanine (DHA) form and is structurally analogous to HvThi4 Cys165 as indicated in pink.

To further compare THI4 family proteins, the 3D-stuctures of the HVO_0665, TK0434 and MA_2851 homologs were predicted by Phyre2-based fold-recognition and model building (see Methods for details). The resulting 3D-models were overlaid with the X-ray crystal structures of THI4p of *S. cerevisiae* (PDB: 3FPZ) and *N. crassa* (PDB: 3JSK) (Figure 2) to identify structurally conserved active site residues using Chimera 1.7 to visualize the models [21]. Conserved active site residues were identified based on analogy to residues shown to be important in ScTHI4p structure and activity [5] as well as residues bound to adenosine diphosphate 5-(beta-ethyl)-4-methyl-thiazole-2-carboxylic acid (AHZ) in the atomic structure of *N. crassa* THI4p. From this analysis, all three archaeal THI4 homologs were found to have close structural similarity to the yeast and fungal enzymes (Figure 2A-C). However, only the HvThi4 protein HVO_0665 (not TK0434 or MA_2851) was found to have residues analogous to those bound to AHZ in the atomic structure of *N. crassa* THI4p as well as the conserved catalytic cysteine of ScTHI4p required for sulfur mobilization to the thiazole ring (Figure 2B).

### Generation of a thiamine auxotroph of *Haloferax volcanii* by deletion of the THI4 homolog

**Figure 3:**
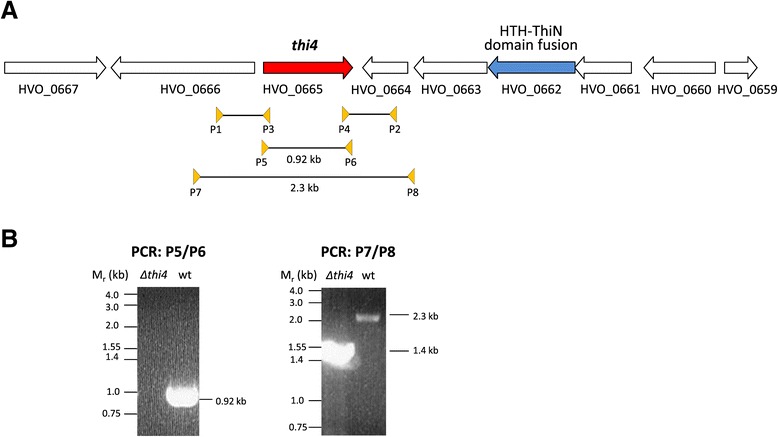
**The** ***Haloferax volcanii thi4*** **gene and its in-frame deletion. (A)** Schematic representation of the *thi4* gene on the genome of *Hfx. volcanii* DS2. HVO_0662 encodes a ThiN homolog with a predicted N-terminal helix-turn-helix (HTH) DNA binding domain. The PCR primer pairs (P1/P2 and P3/P4) used to generate the *thi4* gene deletion and the PCR primer pairs (P5/P6 and P7/P8) used to screen for the *thi4* gene deletion are indicated. **(B)** PCR products generated for the *Δthi4* (*Δhvo_0665*) mutant and parent (H26, wt) strains using primer pairs P5/P6 and P7/P8 as indicated. Size reduction with primer pair P7/P8 and the absence of a signal with primer pair P5/P6 confirm the deletion in the *Δthi4* strain.

To further understand the molecular mechanisms used by archaea with predicted ThiS/ThiF- and THI4-type pathways to synthesize the thiazole ring of thiamine, a genetic strategy was used in which *Hfx. volcanii* strains with targeted deletions of gene homologs in the two predicted pathways were analyzed for growth in the absence of supplied thiamine. Consistent with our metabolic reconstruction results, *Hfx. volcanii* has the metabolic capacity for thiamine biosynthesis as shown by its ability to grow on minimal medium in the absence of an exogenous source of thiamine [23]. *Hfx. volcan*ii is also demonstrated to synthesize key metabolic enzymes that are TPP-dependent including the 2-oxoacid (α-ketoacid): ferredoxin oxidoreductases used to mediate the oxidative decarboxylation of pyruvate and 2-oxoglutarate [24] and three 2-oxoacid dehydrogenases used under nitrate-respirative conditions [25] including one involved in growth on isoleucine [26]. The *ΔubaA* mutant required to analyze the ThiS/ThiF-type pathway was already available from previous study [11]. A *Δthi4* (HVO_0665) mutant strain (NC1011) was generated for this study (Figure 3) using a *pyrE* based pop-in/pop-out strategy similar to generation of the *ΔubaA* mutant (Table 1).

**Figure 4:**
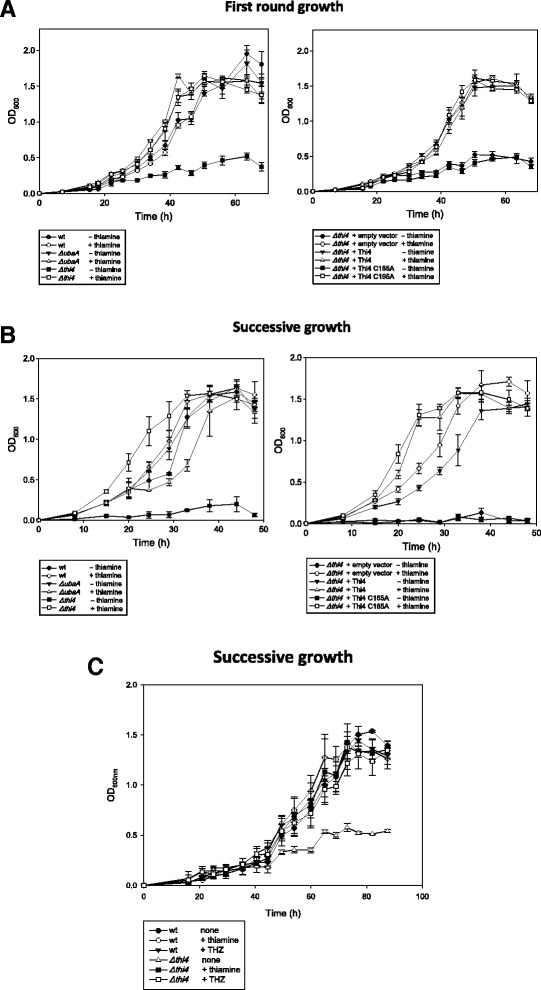
**The** ***Haloferax volcanii*** **THI4 homolog is required for growth in the absence of thiamine and the thiazole moiety 4-methyl-5-(β-hydroxyethyl)thiazole (THZ).** *Hfx. volcanii* strains including H26 parent (wt), HM1052 (*ΔubaA*), NC1011 (*Δthi4*), NC1011-pJAM202c (*Δthi4* + empty vector), NC1011-pJAM2821 (*Δthi4* + HvThi4), and NC1011-pJAM2822 (*Δthi4* + HvThi4 C165A) were grown in GMM supplemented with and without thiamine or THZ as indicated and described in the Materials and Methods. First round **(A)** and successive growth curves **(B and C)** are presented with the mean ± standard deviations shown.

Once generated, the *ΔubaA* and *Δthi4* mutant strains were compared to parent H26 for thiamine-dependent growth. Growth was monitored by optical density (OD_600_) under aerobic conditions on glycerol minimal medium (GMM) with and without the addition of thiamine after transfer from rich medium (Figure 4A; first round growth) and subculture from GMM (Figure 4B; successive growth) (see Methods for details). The *ΔubaA* and parent strains were found to exhibit generation times (G) of 8–10 h and specific growth rates (μ) of 0.03 to 0.04 h^−1^ that were similar and relatively independent of thiamine supplementation (Figure 4A-B). Stationary-phase cultures of these strains (*ΔubaA* and parent) reached comparable cell densities of 1.5 – 1.7 OD_600_ units (Figure 4A-B). Likewise, the *Δthi4* mutant strain displayed growth patterns similar to the parent when cultured on medium supplemented with thiamine. However, little to no growth was observed for the *Δthi4* mutant when successively cultured in the absence of thiamine (Figure 4A-B). We classify the *Δthi4* mutant as a thiamine auxotroph according to its growth behavior. In addition to thiamine, the growth defect of the *Δthi4* mutant observed in the absence of thiamine could be overcome by supplementation of growth medium with the thiazole moiety 4-methyl-5-(β-hydroxyethyl)thiazole (THZ) (Figure 4C). Thus, the *Δthi4* mutant strain is able to salvage the THZ thiazole moiety for re-use in thiamine synthesis and presumably phosphorylate this to THZ-P for condensation with HMP-PP (4-amino-hydroxymethyl-2-methylpyrimidine pyrophosphate), a purine derivative that is synthesized by a separate branch. These results provide strong evidence that synthesis of the thiazole moiety of thiamine (not the HMP-PP) is disrupted in the *Δthi4* mutant strain and that *Hfx. volcanii* uses a Thi4-type (and not a ThiS/ThiF-type) mechanism for sulfur incorporation into the thiazole ring.

**Figure 5:**
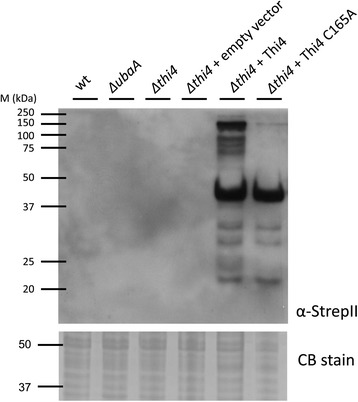
**The THI4 homolog (HvThi4) and its C165A variant are synthesized with C-terminal StrepII tags as stable derivatives in** ***Haloferax volcanii*** **.** Production of the HvThi4-StrepII and HvThi4 C165A-StrepII proteins in the *trans* complemented strains (NC1011-pJAM2821 and -pJAM2822, respectively) was detected by anti-StrepII Western blotting (0.065 OD_600_ units of cells per lane). Equivalent protein loading was assessed by staining blots with Ponceau Red S (not shown) and parallel SDS-PAGE gels with Coomassie Blue R-250 (CB stain, 37–50 kDa range of gel shown).

We next wanted to confirm that the thiamine auxotrophy of the *Hfx. volcanii Δthi4* mutant was directly attributed to the *thi4* deletion by complementation analysis. We also wanted to analyze the influence of the conserved cysteine residue (Cys165) on HvThi4 activity. To answer these questions, the *Δthi4* mutant strain was transformed with plasmids encoding wild-type and C165A variant forms of HvThi4, and the transformed strains were compared to *Δthi4* and parent H26 for growth on thiamine minus medium (Figure 4A-B). The plasmid expressed forms of HvThi4 contained a C-terminal StrepII tag to facilitate detection of the protein produced in *Hfx. volcanii* by anti-StrepII Western blotting. Growth of the *Δthi4* mutant strain *trans* complemented with a wild-type copy of *thi4* was similar to the parent H26, revealing that the thiamine auxotrophy is due to the absence of *thi4* and not a distal effect of the in-frame deletion of this gene. Addition of the C-terminal StrepII tag had no apparent impact on this *trans* complementation suggesting HvThi4-StrepII is activated. In contrast to wild type HvThi4, the gene encoding the HvThi4 C165A variant was unable to *trans* complement the *Δthi4* mutation. Western blotting against the C-terminal StrepII tag of HvThi4 (Figure 5) confirmed that the wild-type and C165A variant forms of HvThi4 were produced as proteins at similar levels in the cell suggesting that the differences in complementation are not due to HvThi4 protein levels and instead are based on enzyme activity. Thus, the conserved active site cysteine (Cys165) of HvThi4 is likely required for catalytic activity similarly to THI4p Cys205 of yeast.

Based on these results, we suggest that Thi4 functions as a suicide thiamine thiazole synthase in *Hfx. volcanii* similarly to yeast [5]. If so, build-up of inactivated HvThi4 in its dehydroalanine form is anticipated. When expressed in *Hfx. volcanii*, the primary isoform of HvThi4 and its C165A variant that accumulated in cells was observed at 40 kDa, which is in relatively close agreement to the theoretical molecular mass of 33.6 kDa for an acidic protein (pI 4.43) likely to bind less SDS than the protein standards used to estimate its molecular mass [27]. However, at least four protein bands of 60 to 150 kDa were detected for the wild type form of HvThi4 that were not observed for the C165A variant. If HvThi4 uses a suicide mechanism, the “dehydroalanine” form of HvThi4 Cys165 generated after sulfur transfer to the thiazole ring precursor would be inactivate and, thus, the protein ‘reagent’ may be susceptible to aggregation or some type of post-translational modification that could facilitate its elimination from the cell. The sampylation system may fulfill this role in archaea by covalently attaching ubiquitin-like SAMP moieties to Thi4 and targeting it for proteasome-mediated degradation [28]. Alternatively, *Hfx. volcanii* cells may have a mechanism to minimize overproduction of thiamine through modification of Thi4.

## Conclusions

**Figure 6:**
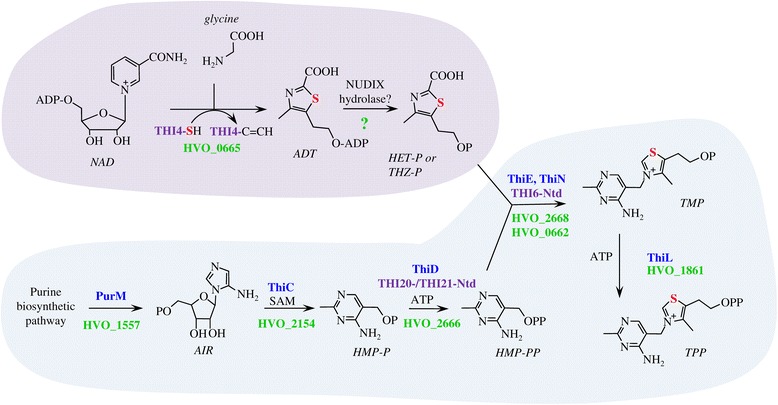
**The pathway for** ***de novo*** **biosynthesis of thiamine in the archaeon** ***Haloferax volcanii*** **is an apparent hybrid of bacterial and yeast pathways.** Yeast and bacterial like steps are shaded by purple and blue, respectively, with associated enzymes of thiamine biosynthetic indicated in text with similar color coding. *Hfx. volcanii* homologs are indicated by gene locus tag in green. Question mark (?) designated enzyme is yet unassigned. THI4-SH specifies the catalytic cysteine side chain. THI4-C = CH indicates the dehydroalanine form of the enzyme after sulfur transfer. The sulfur atom associated with formation of the thiazole ring is highlighted in red. In the *Hfx. volcanii* model for thiamine biosynthesis, the thiazole moiety of thiamine (4-methyl-5-(β-hydroxyethyl)thiazole phosphate; HET-P or THZ-P) is generated by a yeast-like mechanism. HvThi4 (HVO_0665) converts nicotinamide adenine dinucleotide (NAD) and glycine to ADP-thiazole (ADT), which predicted to be hydrolyzed to THZ-P by a yet to be identified NUDIX-type hydrolase [29]. The remaining steps of thiamine biosynthesis are related to bacterial systems. In particular, the PurM-like AIR synthetase HVO_1557 is predicted to form 5-amino-1-(5-phospho-D-ribosyl)imidazole (synonym 5-aminoimidazole ribotide; AIR), which serves as a substrate for a ThiC-like S-adenosyl-methionine (SAM)-dependent HMP-P synthase (HVO_2154) in the generation of 4-amino-hydroxymethyl-2-methylpyrimidine phosphate (HMP-P). Once generated, HMP-P is phosphorylated by a bacterial ThiD-type HMP-P kinase (HVO_2666) which is also conserved in the N-terminal domains (Ntd) of yeast THI20 and THI21. Thiamine-phosphate synthase homologs of the ThiE-type (HVO_2668) and ThiN-type (HVO_0662) are predicted to condense THZ-P with HMP-PP to form thiamine monophosphate (TMP). TMP is then phosphorylated to TPP by a proposed bacterial ThiL-type thiamine-monophosphate kinase (HVO_1861).

Here we resolve the enigma of how sulfur is incorporated into the thiazole ring of thiamine in archaea such as *Hfx. volcanii* and propose a model in which bacterial related enzymes are used for synthesis of the pyrimidine moiety (HMP-PP) and eukaryotic enzymes are used for formation of the thiazole derivative (HET-P or THZ-P) of thiamine (Figure 6). While genomic reconstruction suggested *Hfx. volcanii* used two distinct mechanisms for sulfur incorporation into the thiazole ring, *in vivo* analysis revealed that only the yeast Thi4-type (and not the bacterial ThiF-ThiS) system was required. In particular, the conserved catalytic cysteine residue (Cys165) of the *Hfx. volcanii* THI4p homolog (HvThi4) was found to be essential in the *trans* complementation of a *Δthi4* mutant for growth in the absence of thiamine or THZ. Cluster analysis of THI4p homologs revealed a clear separation of archaeal homologs with a conserved active site cysteine residue, such as HvThi4, from those having a histidine residue in its place (the latter of which are implicated in functions distinct from thiamine biosynthesis [6,7,22]). Based on this distinction, we propose a yeast-type THI4p mechanism, which uses an active site cysteine residue for sulfur incorporation into the thiazole ring of thiamine, is relatively widespread in archaea including halophilic archaea of the phylum Euryarchaeota and ammonium oxidizing archaea of the phylum Thaumarchaeota.

### Availability of supporting data

All supporting data are included as an additional Supplementary Materials file deposited in LabArchives, LLC under the dataset link DOI:10.6070/H4959FJX.

## Additional file

Additional file 1: **Supplementary Materials.**
