# Supplementary material for: Conserved active site cysteine residue of archaeal THI4 homolog is essential for thiamine biosynthesis in Haloferax volcanii
Source: BMC Microbiol. 2014 Oct 28;14:260. doi: 10.1186/s12866-014-0260-0 (PMC4215014; doi:10.1186/s12866-014-0260-0)
Supplement: Supplementary file 1 — Supplementary Materials. [file 12866_2014_260_MOESM1_ESM.pdf]

# Supplementary Materials for Hwang *et al*. (2014)

# Supplementary text

**Thiamine pyrophosphate biosynthesis homologs of *Hfx. volcanii* identified by comparison to bacterial and yeast pathways.** Reconstruction of thiamine biosynthesis in halophilic archaea has been previously reported [1, 2]. Here we update the annotation of the complete genome sequence of *Hfx. volcanii* DS2 [3] with respect to homologs related to bacterial and eukaryotic enzymes of *de novo* and salvage pathways used for thiamine pyrophosphate (TPP) biosynthesis. For details and references to support biological function of these enzymes see Fig. S1 and Table S1-2. Conserved homologs were identified based on clustering to proteins classified with thiamine biosynthetic pathways in InterPro [4] and NCBI Conserved Domain Databases (CDD) [5] as well as 3D structural homology modeling using Phyre2 [6].

***De novo biosynthesis of thiamine***. *De novo* biosynthesis of thiamine (Fig. S1A-B) involves separate synthesis of two intermediate molecules: 4-amino-hydroxymethyl-2-methylpyrimidine pyrophosphate (HMP-PP) and 4-methyl-5-(β-hydroxyethyl)thiazole phosphate (HET-P or THZ-P). Once formed, HMP-PP and HET-P are condensed to generate thiamine monophosphate (TMP). TMP is then phosphorylated to TPP.

**(a) *HMP-PP synthesis.*** In bacteria, HMP-PP synthesis is described as starting from 5-amino-1-(5-phospho-D-ribosyl)imidazole (AIR), an intermediate of purine biosynthesis that is generated by PurM. AIR is converted to 4-amino-hydroxymethyl-2-methylpyrimidine phosphate (HMP-P) in a complex rearrangement by the radical SAM enzyme ThiC, the key enzyme for the bacterial pathway. HMP-P is phosphorylated by ThiD leading to HMP-PP, which is one substrate of the condensation reaction. In yeast, the key enzyme THI5p mediates the synthesis of HMP-P from histidine and pyridoxal-phosphate (PLP). HMP-P is subsequently phosphorylated to HMP-PP by THI21 and THI20 paralogues, which have N-terminal domains related to bacterial ThiD that are important for function. As can be seen by the provided ORF codes, *Hfx. volcanii* encodes a homolog to each of these enzymes with exception of the key enzyme THI5. THI5 pyrimidine synthase family (IPR027939) members are found in some archaea and bacteria but not *Hfx. volcanii.*

**(b) *HET-P synthesis***. HET-P is generated in bacteria from iminoacetate (derived from glycine or tyrosine), 1-deoxy-D-xylulose 5-phosphate (DXP), and a sulfur atom in activated form. These intermediates are condensed to a tautomer of hydroxyethylthiazole phosphate (cTHZ*-P) by ThiG and then tautomerized by TenI to HET-P, the other substrate of the condensation reaction. Generation of iminoacetate from glycine requires ThiO and from tyrosine requires ThiH (not shown). As can be seen by the absence of ORF codes, there are no homologs to any of these enzymes (ThiG, ThiO, TenI, or ThiH) in *Hfx. volcanii*. Note that the description of sulfur activation and eukaryotic synthesis of HET-P is described in later sections.

**(c) *Condensation of HMP-PP and HET-P.*** Two alternate enzymes (ThiE and ThiN) catalyze the condensation of HMP-PP and HET-P to thiamine monophosphate (TMP) in bacteria. Yeast encode a bifunctional THI6p that has a ThiE-type TMP synthase fused to a C-terminal ThiM domain, which salvages HET-P by phosphorylation of 4-methyl-5-(β-hydroxyethyl)thiazole (THZ). *Hfx. volcanii* genome codes for both ThiE- and ThiN-type TMP synthases with the ThiN homolog (HVO_0662) fused to an N-terminal helix-turn-helix (HTH) DNA binding domain (Fig. S2) suggesting it may function in transcriptional regulation of the pathway. After condensation, TMP is phosphorylated by ThiL (bacteria) or hydrolyzed and then pyrophosphorylated by THI80p (yeast) to the final product thiamine pyrophosphate (TPP). Similarly to other archaea, *Hfx. volcanii* codes for a ThiL (not a THI80p) homolog.

***Thiamine salvage***. Microbes have evolved transporters and kinases to uptake and salvage thiamine derivatives present in the environment (Fig. 1C). In bacteria, an ABC-type transporter (ThiBPQ) is used for the uptake of thiamine and TPP and appears conserved in *Hfx. volcanii.* The putative transmembrane protein HVO_0023 of the UPF0118 superfamily may associate with this ABC-type thiamine transporter based on genome neighborhood linkage. *Hfx. volcanii* is also predicted to uptake thiamine precursors by a symport mechanism based on coding sequence overlap of HVO_B0379 (PtuP2, a $Na^+$/solute symporter homolog) with HVO_B0380 (TenA2, a homolog of bacterial TenA and yeast THI20 C-terminal domain thiaminase II enzymes). Thiaminase II cleaves thiamine related compounds including those generated by YlmB-mediated deformylation to generate hydroxymethylpyrimidine (HMP). HMP is successively phosphorylated through a series of ThiD mediated kinase reactions to synthesize HMP-PP. *Hfx. volcanii* has homologs to all of these enzymes (TenA, YlmB and ThiD) suggesting it can synthesize HMP-PP by a salvage pathway. *Hfx. volcanii* also appears to salvage HET-P through phosphorylation of THZ based on identification of the ThiM homolog HVO_2667. Thiamine pyrophosphokinase (TPK) enzymes of the IPR006282 family that convert thiamine to TPP were restricted to bacteria and eukaryotes with no homologs identified in *Hfx. volcanii* or other archaea.

***Sulfur activation for the thiazole ring***. In the bacterial pathway, sulfur is provided for ThiG in an activated form, as thiocarboxylate on the C-terminal glycine of the carrier protein ThiS. Generation of this thiocarboxlate starts with activation of ThiS by adenylation, which is catalyzed by ThiF. The adenylate is then exchanged against a sulfur atom provided by ThiI. Homologs for all of these proteins are identified in *Hfx. volcanii*. ThiS has a ubiquitin-fold, and its *Hfx. volcanii* structural homologs (SAMP1, HVO_2619; SAMP2, HVO_0202; SAMP3, HVO_2177 with a corrected start codon to result in a 92 aa protein) were shown to be covalently attached to target proteins in a process called sampylation [7, 8]. SAMP1 and SAMP2 were also shown to be involved in sulfur chemistry, SAMP1 participating in biosynthesis of molybdopterin while SAMP2 participates in thiolation of tRNA [9]. *Hfx. volcanii* has only a single E1-type enzyme (UbaA, HVO_0558) which belongs to the ThiF/MoeB/HesA family and adenylates all three SAMPs based on its requirement for SAMP function [8, 9]. Thus, we have the rare opportunity to determine if any of the SAMPs are involved in sulfur chemistry of thiamine biosynthesis by analyzing a *ΔubaA* strain (this study).

An eventual involvement of the This-ThiF homologs, SAMP(1-3)-UbaA, would require sulfur transfer from ThiI (HVO_1651). However, *Salmonella enterica* ThiI provides sulfur for thiamine biosynthesis via its rhodanese domain, a domain also occurring in the *E. coli* ortholog [10]. This rhodanese domain is found in a minority of the ThiI homologs and is missing from HVO_1651, making involvement on HVO_1651 in thiamine biosynthesis rather unlikely. In addition to thiamine biosynthesis, *S. enterica* ThiI is also involved in thiolation of tRNA, a function which requires only the two N-terminal domains [10]. Thus, nearly all of the proteins named “thiamine biosynthesis protein ThiI” in the databases are concluded to be completely unrelated to thiamine biosynthesis but instead are involved in generation of the modified tRNA base 4-thiouridine [11]. Similarly to the methanogen homolog MMP1354, this is also the likely function of HVO_1651 as we find it is not required for growth of *Hfx. volcanii* in the absence of thiamine (data not shown).

# Supplementary Tables

**Suppl. Table S1.** *Haloferax volcanii* DS2 gene homologs of thiamine (vitamin B1) metabolism and transport[a,b].

| Gene | *Hfx. volcanii* ORF locus tag, aa | *Hfx. volcanii* ORF GI, UniProtKB | Thiamine biosynthesis function | Highly conserved domain(s), E value | *Hfx. volcanii* ORF aCOG / COG | Evidence (organism, ref.) |
|---|---|---|---|---|---|---|
| *thiQ* | HVO_0020, 361 aa | GI:292654197, UniProtKB:D4GYL5 | ABC thiamine transporter ATPase | COG3842: PotA, ABC-type spermidine/putrescine transport systems, ATPase components, 1.03e-123 | arCOG00177, COG3842 | B, *S. typhimurium* ABC transporter (ThiBPQ) required for transport of thiamine and TPP [12] |
| *thiP* | HVO_0021, 573 aa | GI:292654198, UniProtKB:D4GYL6 | ABC thiamine transporter permease | COG1178: ThiP, ABC-type Fe3+ transport system, permease component, 2.55e-65 | arCOG00163, COG1178 | |
| *thiB* | HVO_0022, 378 aa | GI:292654199, UniProtKB:D4GYL7 | ABC thiamine transporter substrate-binding protein | COG4143: ABC-type thiamine transport system, periplasmic component, 1.80e-85 | arCOG00226, COG4143 | |
| *thiF*<br>--[c] | HVO_0558 (UbaA), 270 aa | GI:292654724, UniProtKB:D4GSF3 | Thiazole biosynthesis adenylyl-transferase | cd00757: ThiF/MoeB/HesA family, 3.70e-102 | arCOG01676, COG0476 | B, *E. coli* ThiF [13]; |
| *thiN* | HVO_0662, 299 aa | GI:292654826, UniProtKB:D4GSS2 | TMP synthase (ThiN) with N-terminal helix-turn-helix (HTH) domain | pfam10120: Putative aldolase; Members of this family of archaeal and bacterial proteins are likely to be aldolases, 1.12e-47 | arCOG00021, COG1992 | B, *T. maritima* ThiN domain [14]<br>A, *P. calidifontis* ThiN domain [15] |
| *THI4* | HVO_0665, 307 aa | GI:292654829, UniProtKB:D4GSS5 | Suicide thiamine thiazole synthase | PRK04176: ribulose-1,5-biphosphate synthetase, provisional, 1.22e-124 | arCOG00574, COG1635 | E, *S. cerevisiae* THI4p [16] |
| *thiI*<br>--[d] | HVO_1651, 391 aa | GI:292655800, UniProtKB:D4GZL6 | Thiamine/ thiouridine biosynthesis protein | COG0301: ThiI, thiamine biosynthesis ATP pyrophosphatase, 6.12e-112 | arCOG00038, COG0301 | B, *S. enterica* ThiI rhodanese-like (RHD) domain [10, 17] |
| *thiL* | HVO_1861, 297 aa | GI:292655996, UniProtKB:D4GSW4 | TMP kinase | cd02194: ThiL (TMP kinase) plays a dual role in de novo biosynthesis and in salvage of exogenous thiamine, 2.60e-66 | arCOG00638, COG0611 | B, *S. typhimurium* ThiL [19] |
| *thiC* | HVO_2154, 482 aa | GI:292656282, UniProtKB:D4GV87 | HMP-P synthase | pfam01964: ThiC within the thiamine biosynthesis operon. ThiC participates in the formation of HMP-P from AIR, an intermediate in the de novo pyrimidine biosynthesis, 0e+00 | arCOG02741, COG0422 | B, *E. coli* ThiC [20, 21] and *B. subtilis* ThiA (ThiC homolog) [22] |
| *purM* | HVO_1557, 324 aa | GI: 292655707, UniProtKB:D4GYY6 | AIR synthase | IPR004733, phosphoribosylformyl-glycinamidine cyclo-ligase family (HVO_1557 is only *Hfx. volcanii* member) | arCOG00636, COG0309 | B, *E. coli* PurM [23, 24] |
| *thiS*<br>--[e] | HVO_2619 (SAMP1), 87aa<br>HVO_0202 | GI: 292656738, UniProtKB:D4GUF6<br>GI: 292654382, | Thiamine biosynthesis sulfur carrier protein | Ubiquitin-fold superfamily, small ubiquitin-fold archaeal | arCOG00536, COG1977<br>arCOG00535, | B, *B. subtilis* and *E. coli* ThiS [25, 26] |

| | | | | | | |
|---|---|---|---|---|---|---|
| | (SAMP2), 66 aa<br>HVO_2177 (SAMP3) M15-G106, 92 aa | UniProtKB:D4GZE7<br>GI: 292656305, UniProtKB:D4GVB0 | | modifier proteins, 1.34e-15 (SAMP1), 4.37e-06 (SAMP2) and 1.25e-17 (SAMP3) | COG2104<br>arCOG00536, COG1977 | |
| *thiD* | HVO_2666, 279 aa | GI:292656785, UniProtKB:D4GV38 | HMP and HMP-P kinase (ThiD) (or synonym pyridoxine, pyridoxal, and pyridoxamine kinase, PdxK) | cd01169: HMP and HMP-P kinase, 4.66e-81 | arCOG00020, COG0351 | B, *B. subtilis* ThiD (PdxK) [31]<br>E, *S. cerevisiae* THI21p and THI20p (ThiD domains) [32, 33] |
| *thiM* | HVO_2667, 298 aa | GI:292656786 UniProtKB:D4GV40 | THZ kinase | cd01170: THZ kinase, catalyzes the phosphorylation of the hydroxyl group of THZ, 1.02e-47 | arCOG00019, COG2145 | B, *E. coli* ThiM [34]<br>E, *A. thaliana* THIMp (potential origins from mitochondria/ chloroplast) [35] |
| *thiE* | HVO_2668, 214 aa | GI:292656787, UniProtKB:D4GV42 | TMP synthase | cd00564: TMP synthase, TenI, 6.21e-47 | arCOG01089, COG0352 | B, *B. subtilis* ThiE [36, 37] |
| *tenA* | HVO_B0381, 221 aa<br>HVO_B0380, 261 aa | GI:292494313, UniProtKB:D4GQ27<br>GI:292494312, UniProtKB:D4GQ26 | Thiaminase II | Thiaminase II (IPR027574); COG0819: TENA, THI-4, PQQC family, 1.34e-73 and 1.37e-36 | arCOG01128, COG0819 | B, *B. subtilis* TenA [38, 39]<br>E, *S. cerevisiae* THI20p [40] |
| *ylmB* | HVO_B0002, 385 aa | GI:292493940, UniProtKB:D4GP03 | AMPF deformylase? | Acetylornithine deacetylase/ succinyl-diaminopimelate desuccinylase family (IPR010182) | arCOG01107, COG0624 | B, *B. subtilis* BsYlmB [39]; Predicted AMPF deformylase based only on co-clustering with BsYlmB to IPRO10182 and genomic linkage with HVO_B0381 (*tenA1*) and HVO_B0380 (*tenA2*) |
| SSSF protein | HVO_B0379, 509 aa | GI:292494311, UniProtKB:D4GQ25 | Sodium:solute symporter family (SSSF) (IPR001734) protein | cl00456: SLC5-6-like_sbd Superfamily, 3.70e-87 | arCOG01319, COG0591 | Predicted uptake of thiamine-related compounds based on clustering to SSSF and coding sequence overlap with HVO_B0380 (*tenA2*) |
| -- | HVO_0023, 296 aa | GI:291371158 UniProtKB:D4GYL8 | transmembrane protein associated with ThiBPQ ABC-type transporter of thiamine salvage | cl00465: UPF0118 superfamily. Transmembrane region of unknown function found in putative permeases and predicted transmembrane proteins, 5.89e-10 | arCOG02642, COG0628 | Predicted association with ThiPBQ transporter function based on gene synteny and conservation of a permease-like transmembrane region |
| *THI5* | -- | -- | HMP-P synthesis from histidine and PLP | pfam09084: THI5-like family. THI5 (NMT1) proposed to be required for the biosynthesis of the pyrimidine moiety of thiamine. Regulated by thiamine. | -- | E, *S. cerevisiae* THI5p [41] |
| *thiG* | -- | -- | thiazole synthase | IPR008867: thiazole biosynthesis family | -- | B, *B. subtilis* ThiG [26] |
| *tenI* | -- | -- | thiazole tautomerase | cd00564: TenI TMP synthase | -- | B, *B. subtilis* TenI [42] |
| *thiO* | -- | -- | glycine oxidase | TIGR02352: thiamin_ThiO glycine | -- | B, *B. subtilis* ThiO [43, 44] |

| | | | | | | |
|---|---|---|---|---|---|---|
| | | | | oxidase family; pfam01266: DAO FAD dependent oxidoreductase family | | |
| *thiH* | -- | -- | tyrosine lyase | cd01335: Radical_SAM superfamily; PRK09240: thiamine biosynthesis protein ThiH | -- | B, *E. coli* ThiH [45, 46] |
| *THI6* | -- | -- | bifunctional TMP diphosphorylase / hydroxyethyl-thiazole kinase | N-terminal domain COG0352: ThiE, TMP synthase; C-terminal domain COG2145: ThiM, hydroxyethylthiazole kinase, sugar kinase family | -- | E, *S. cerevisiae* THI6p [47, 48] |
| *THI80* | -- | -- | thiamine pyrophospho-kinase | COG1564: THI80 Thiamine pyrophosphokinase; cd07995: Thiamine pyrophosphokinase (TPK, EC:2.7.6.2) | -- | E, *S. cerevisiae* Thi80 [49] |
| *tpk* | -- | -- | thiamine pyrophospho-kinase | (IPR006282) Thiamine pyrophosphokinase (EC:2.7.6.2); TPK catalytic domain (IPR007371) | -- | E, *S. cerevisiae* TPK [50]; B, *S. aureus* TPK [51] |

[a] --, *Hfx. volcanii* gene homologs not predicted or not applicable. COG and aCOG classification according to Wolf *et al.* [52].

[b]Abbreviations: A, Archaea; B, Bacteria; E, Eukarya; ADP-thiazole (ADT); deoxy-D-xylulose 5-phosphate (DXP); 5-aminoimidazole ribotide or 5-amino-1-(5-phospho-D-ribosyl)imidazole (AIR); S-adenosyl-methionine (SAM); thiazole tautomer (R,Z)-2-(2-carboxy-4-methylthiazol-5(2H)-ylidene)ethyl phosphate (cTHZ*-P); pyridoxal 5'-phosphate (PLP), nicotinamide adenine dinucleotide (NAD); 4-methyl-5-(β-hydroxyethyl)thiazole (THZ); 4-methyl-5-(β-hydroxyethyl)thiazole phosphate (THZ-P; synonym 4-methyl-5-(β-hydroxyethyl)thiazole phosphate, HET-P); thiamine monophosphate (TMP); thiamine pyrophosphate (TPP); hydroxymethylpyrimidine (HMP); 4-amino-hydroxymethyl-2-methylpyrimidine pyrophosphate (HMP-PP); 4-amino-hydroxymethyl-2-methylpyrimidine phosphate (HMP-P); N-[(4-amino- 2-methylpyrimidin-5-yl)methyl]formamide (AMPF); 4-amino-5-aminomethyl-2-methylpyrimidine (AAMP); *Salmonella typhimurium* (*S. typhimurium*); *Escherichia coli* (*E. coli*); *Thermotoga maritima* (*T. maritima*); *Pyrobaculum calidifontis* (*P. calidifontis*); *Saccharomyces cerevisiae* (*S. cerevisiae*); *Salmonella enterica* (*S. enterica*); *Bacillus subtilis* (*B. subtilis*); *Haloferax volcanii* (*Hfx. volcanii*); *Staphylococcus aureus* (*S. aureus*); *Arabidopsis thaliana* (*A. thaliana*).

[c]While UbaA shares 39% amino acid identity (over a query coverage 90%) with *E. coli* ThiF, UbaA is not required for thiamine biosynthesis (this study) and instead functions with the ubiquitin-fold SAMPs in the formation of ubiquitin-like isopeptide bonds, the thiolation of tRNA, and the biosynthesis of molybdopterin (MPT) [8, 9].

[d]HVO_1651 is related to ThiI but devoid of the rhodanese domain (RHD). HVO_1651 is not required for thiamine biosynthesis (this study) and likely functions in tRNA modification based on analogy to methanogens [18].

[e]*Hfx. volcanii* SAMPs are Ub-fold proteins structurally related to ThiS [28-30] and function with UbaA in sulfur transfer and protein modification [8, 9, 27]. However, SAMPs do not appear to be linked with thiamine metabolism based on analysis of UbaA (this study).

**Suppl. Table S2.** Yeast THI4p active site cysteine (Cys205) is conserved among select archaeal Thi4 homologs[a].

| | |
|---|---|
| **Eukaryotes** | |
| P32318_Saccharomyces cerevisiae ScTHI4p | VTQAHGTQCCMDPNVIELAG |
| Q38814_ Arabidopsis thaliana AtTHI4p | VAQNHHTQSCMDPNVMEAKI |
| **Euryarchaeota – Haloarchaea** | |
| D4GSS5_Haloferax volcanii (HVO_0665) | VHALPRELTCVDPIAVESDL |
| B0R884_Halobacterium salinarum R1 | VHSLPRELTCVDPIALEADV |
| B9LWD6_Halorubrum lacusprofundi | VHALPRELTCVDPIAVESEL |
| C7NQF3_Halorhabdus utahensis | VHALPREITCVDPIAVESKL |
| C7NVN8_Halomicrobium mukohatae | VHALPREITCVDPIAVEADL |
| D2RQL7_Haloterrigena turkmenica | VHALPREITCVDPIAVEADL |
| D3SXN1_Natrialba magadii | VHALPREITCVDPIAVEADL |
| D8J722_Halalkalicoccus jeotgali | VHALPREITCVDPIAVEADL |
| E4NSK7_Halogeometricum borinquense | VHALPRELTCVDPVAVESDL |
| E7QU08_Haladaptatus paucihalophilus | VHALPRELTCVDPIAVESDL |
| F7PLY7_Halorhabdus tiamatea | VHALPREITCVDPIAVESDL |
| F8D479_Halopiger xanaduensis | VHALPREITCVDPIAVEADL |
| G0HX18_Haloarcula hispanica | VHALPREITCVDPIAVEADL |
| G0LHM9_Haloquadratum walsbyi DSM16854 | VHALPRELTCVDPVAVESDL |
| G2MNC9_halophilic archaeon DL3 | VHALPREITCVDPVAVEADL |
| G4G9D1_Natronobacterium gregoryi | VHALPREITCVDPIAVEADL |
| G4GE57_Natrinema pellirubrum | VHALPREITCVDPIAVEADL |
| Q18KP1_Haloquadratum walsbyi DSM16790 | VHALPRELTCVDPVAVESDL |
| Q3IMI0_Natronomonas pharaonis | VHALPREITCVDPIAVEADL |
| Q3V7Z9_Haloarcula marismortui | VHALPREITCVDPIAVEADL |
| Q9HMC7_Halobacterium salinarum NRC-1 | VHSLPRELTCVDPIALEADV |
| **Euryarchaeota – select species of methanogens and pyrococci** | |
| F4HJT0_Pyrococcus sp. (strain NA2) | VSALPRQITCVDPIALESKI |
| F8AJA7_Pyrococcus yayanosii (strain CH1) | VSALPRQITCVDPIALESKI |
| F6D358_Methanobacterium sp. strain SW | VSALPRAITCVDPVSIESKI |
| F0T6Y7_Methanobacterium sp. (strain AL-21) | VSALPRAITCVDPVALESKI |
| H8I7V6_Methanocella conradii | VSSLPREITCVDPIAIESKV |
| K2QAU0_Methanobacterium formicicum | VSALPRAITCVDPVALESKI |
| **Thaumarchaeota** | |
| A0RUI0_Cenarchaeum symbiosum strain A | VSALPRNITCVDPIALEAKM |
| A9A485_Nitrosopumilus maritimus strain SCM1 | VSALPRNITCVDPVAFEAKI |
| B3T7X2_uncultured marine crenarchaeote | VSALPRNITCVDPIALEAKM |
| F3KID9_Candidatus Nitrosoarchaeum limnia | VSALPRNITCVDPIALEAKM |
| F9CVY3_Candidatus Nitrosoarchaeum koreensis | VSALPRNITCVDPIALEAKM |
| K0B3D5_Candidatus Nitrosopumilus koreensis AR1 | VSALPRNITCVDPVAFEAKV |
| **Crenarchaeota - Aeropyrum** | |
| Q9Y9Z0_Aeropyrum pernix | VQGLPRQITCVDPVGLRAEY |
| **Archaea - with 'histidine -containing' THI4p homologs** | |
| Q58018_Methanocaldococcus jannaschii(MJ0601) | IERAG---LHIDPLTIRSKV |
| Q5JD25_Thermococcus kodakaraensi (TK0434) | VMMTG---LHVDPLTVEAKF |
| Q8TM19_Methanosarcina acetivorans (MA_2851) | VTTQR---LHVDPLMIRTKL |
| A0B880_Methanosaeta thermophila | ADMAG---IHVDPLAIRARV |
| A1RW13_Pyrobaculum islandicum | IQMSG---SHVDPLYTQAKA |
| A2BJG4_Hyperthermus butylicus | VVEAG---WHVDPIYIEARA |
| A2SQ47_Methanocorpusculum labreanum | VVREG---LHVDPLSFRAKI |
| A3CXS4_Methanoculleus marisnigri | VDMAG---LHVDPLTMACTC |
| A3DKK5_Staphylothermus marinus | IFEAG---WHVDPFYVEAKA |
| A3MWF6_Pyrobaculum calidifontis | IQMSG---MHVDPLYTQAKA |
| A4FWG7_Methanococcus maripaludis | IEKAG---LHIDPLTISAKY |
| A4WKY7_Pyrobaculum arsenaticum | IQMSG---MHVDPLYTMAKA |
| A4YIV7_Metallosphaera sedula | TQMAS---LHVDPLFISAKA |
| A6UPZ7_Methanococcus vannielii | IEKAG---LHVDPITISAKY |
| A6UV59_Methanococcus aeolicus | INKAG---LHIDPLTINAKY |
| A6VGT9_Methanococcus maripaludis | IEKAG---LHIDPLTISAKY |
| A8A9Z0_Ignicoccus hospitalis | IEIAG---LHVDPIFFKSKA |
| A8M926_Caldivirga maquilingens | IQMAG---VHTDPFFIESNA |
| A9A9W1_Methanococcus maripaludis | IEKAG---LHIDPLTISAKY |
| B1L513_Korarchaeum cryptofilum | VLLAG---LHVDPLFIHSRV |
| B1YDX0_Thermoproteus neutrophilus | IQMSG---SHVDPLYTQARA |
| B8D4K8_Desulfurococcus kamchatkensis | VQLSG---LHVDPLFIESKA |

| | |
|---|---|
| C3MQY1_Sulfolobus islandicus | TQMAS---LHVDPIFISAKA |
| C3MWW9_Sulfolobus islandicus | TQMAS---LHVDPIFISAKA |
| C3N6N6_Sulfolobus islandicus | TQMAS---LHVDPIFISAKA |
| C3N749_Sulfolobus islandicus | TQMAS---LHVDPIFISAKA |
| C3NGI6_Sulfolobus islandicus | TQMAS---LHVDPIFISAKA |
| C4KIA7_Sulfolobus islandicus | TQMAS---LHVDPIFISAKA |
| C5A6B0_Thermococcus gammatolerans | VMRTG---LHVDPLTVEARF |
| C7P9G0_Methanocaldococcus fervidus | IEKAG---LHIDPLTIKSKI |
| C9RDQ9_Methanocaldococcus vulcanius | IERAG---LHIDPLTIRSKV |
| D0KSC6_Sulfolobus solfataricus | TQMAS---LHVDPIFISAKA |
| D2PD13_Sulfolobus islandicus | TQMAS---LHVDPIFISAKA |
| D2REC7_Archaeoglobus profundus | TFMSG---LHVDPLVLRSKV |
| D3S3G6_Ferroglobus placidus | VQIAG---LHVDPLMIESKA |
| D3S5Y8_Methanocaldococcus sp. | IERAG---LHIDPLTIRSKV |
| D5E9H9_Methanohalophilus mahii | VEIGR---LHVDPLTIRSRL |
| D5VTM8_Methanocaldococcus infermus | IEKAG---LHVDPLAIESKV |
| D7D7Y4_Staphylothermus hellenicus | IYEAG---WHVDPFYIEANA |
| D7DTD7_Methanococcus voltae | IEKAG---LHVDPITISAKC |
| D7E696_Methanohalobium evestigatum | VSIAN---LHVDPLTIRAKV |
| D9PUB7_Methanothermobacter marburgensis | VEMAG---LHVDPLTVRAGA |
| E0SQR9_Ignisphaera aggregans | VVMSG---LHVDPLFITSRA |
| E1QR50_Vulcanisaeta distributa | VQMAG---VHTDPFFIEAKA |
| E1RE05_Methanoplanus petrolearius | VEMAG---LHIDPLTMRTKV |
| E3GXE6_Methanothermus fervidus | AEMAK---IHVDPLVIKSKF |
| F0NFC6_Sulfolobus islandicus | TQMAS---LHVDPIFISAKA |
| F0NKN1_Sulfolobus islandicus | TQMAS---LHVDPIFISAKA |
| F0QW10_Vulcanisaeta moutnovski | IQMAN---MHTDPFFIEAKA |
| F0T9W5_Methanobacterium sp. strain AL-21 | VQMGG---LHVDPLTVRSKA |
| F2KNT2_Archaeoglobus veneficus | VEMSG---LHIDPMTVACKA |
| F2L134_Thermoproteus uzoniensis | IQMSG---MHVDPLYIQTKA |
| F4B7H4_Acidianus hospitalis | TQMSG---LHVDPLFISAKA |
| F4BUD4_Methanosaeta concilii | AEMAQ---IHVDPLCIRARY |
| F4FXZ8_Metallosphaera cuprina | TQMAA---LHVDPLFISARA |
| F4HLX9_Pyrococcus sp. strain NA2 | VLMTG---LHVDPLTVEAKY |
| F6BCS4_Methanotorris igneus | IEKAG---LHIDPITITAKY |
| F6BDU0_Methanotorris igneus | IEKAG---LHIDPITITAKY |
| F6D5C3_Methanobacterium sp. strain SW | VEMGG---LHVDPLTVRAKA |
| F7XNG4_Methanosalsum zhilinae | VGIGK---LHVDPLTIRSKV |
| F8AI82_Pyrococcus yayanosii | VRMTG---LHVDPLTVEAKF |
| F8AL44_ Methanothermococcus okinawensis | IDKAG---LHIDPLTINAKY |
| G0EFR7_Pyrolobus fumarii 1A | VGIAN---LHVDPLMFEAKA |
| G0HLR3_Thermococcus sp. strain | VMMTG---LHVDPLTVEARF |
| O27657_Methanothermobacter thermautotrophicus | VEMAG---LHVDPLTVRARA |
| O29556_Archaeoglobus fulgidus | VEISG---LHVDPLFLRSRA |
| O59082_Pyrococcus horikoshii | VLMAG---LHVDPLTIEAKY |
| Q12U93_Methanococcoides burtonii | VEIGK---LHVDPLAIRSKV |
| Q2FM60_Methanospirillum hungatei | VEATG---LHVDPLTIGCKM |
| Q46AR1_Methanosarcina barkeri | VTVQR---LHVDPLMIRTKL |
| Q4JAF8_Sulfolobus acidocaldarium | TQMAG---LHVDPVFISAKA |
| Q6LXJ8_Methanococcus maripaludis | IEKAG---LHIDPITISAKY |
| Q8Q0B5_Methanosarcina mazei | VTTQR---LHVDPLMIRTKL |
| Q8TY75_Methanopyrus kandleri | VKAAN---MHVDPLALEAEY |
| Q8U0Q5_Pyrococcus furiosus | VKMTG---LHVDPLTVEAKY |
| Q8ZZM5_Pyrobaculum aerophilum | IQMSG---MHVDPLYTQAKA |
| Q975R0_Sulfolobus tokodaii | TQMAG---LHVDPLFISAKA |
| Q97ZY5_Sulfolobus solfataricus | TQMAS---LHVDPIFISAKA |
| Q9V0J8_Pyrococcus abyssi | VLMTG---LHVDPLTVEAKY |
| **Archaea - with ‘proline-containing’ THI4p homologs** | |
| B5ID81_Aciduliprofundum boonei | -VIGE---LPIDPLSIYAKY |
| B5IDD0_Aciduliprofundum boonei | -VIGE---LPIDPLSIYTKY |
| **Select bacteria** | |
| Q9WZP4_ Thermotoga maritima | VMMTG---LHVDPLTVEAKF |

[a]Residues in analogous position to conserved active site cysteine (Cys205) of ScTHI4p are highlighted (*i.e.*, cysteine residues in red, histidine residues in black, and proline residues in blue). *Hfx. volcanii* HVO_0665 is the THI4p homolog of this study. MJ0601 and MA_2851 are described as a D-ribose-1,5-bisphosphate isomerases [53]. TK0434 is annotated as a putative ribose 1,5-bisphosphate isomerase but was demonstrated to lack this activity *in vitro* [54]. Note that select species of methanogens and pyrococci have two THI4 homologs including one with a conserved active site cysteine and another with a histidine in this position. UniProtKB/Swiss-Prot numbers are listed for each protein sequence. Gaps introduced to optimize multiple amino acid sequence alignment are indicated by -.

# Supplemental Figures

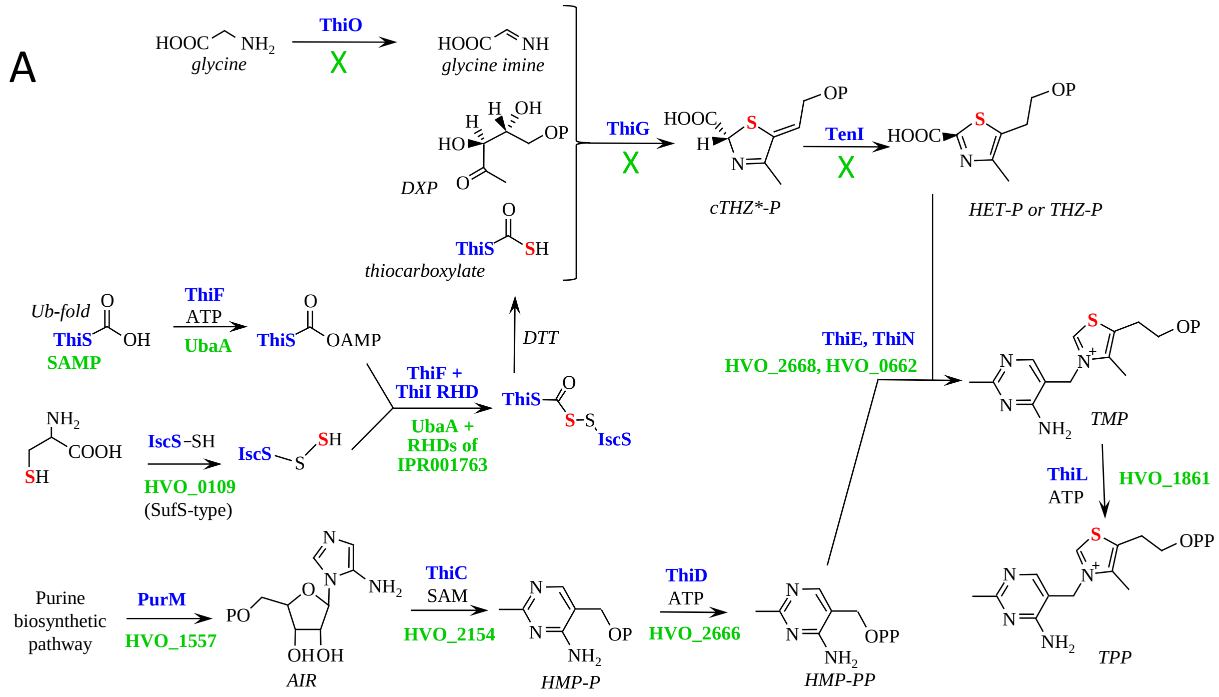


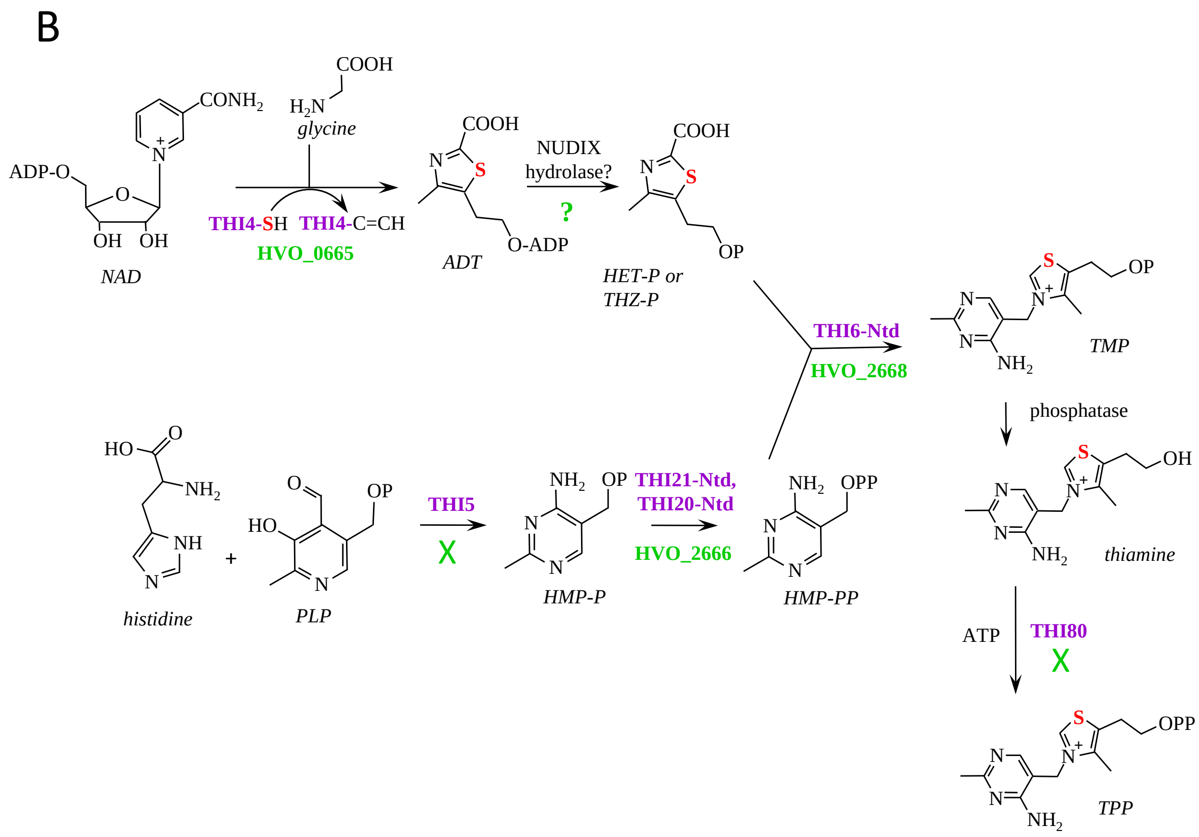


**Figure S1 [cont].**

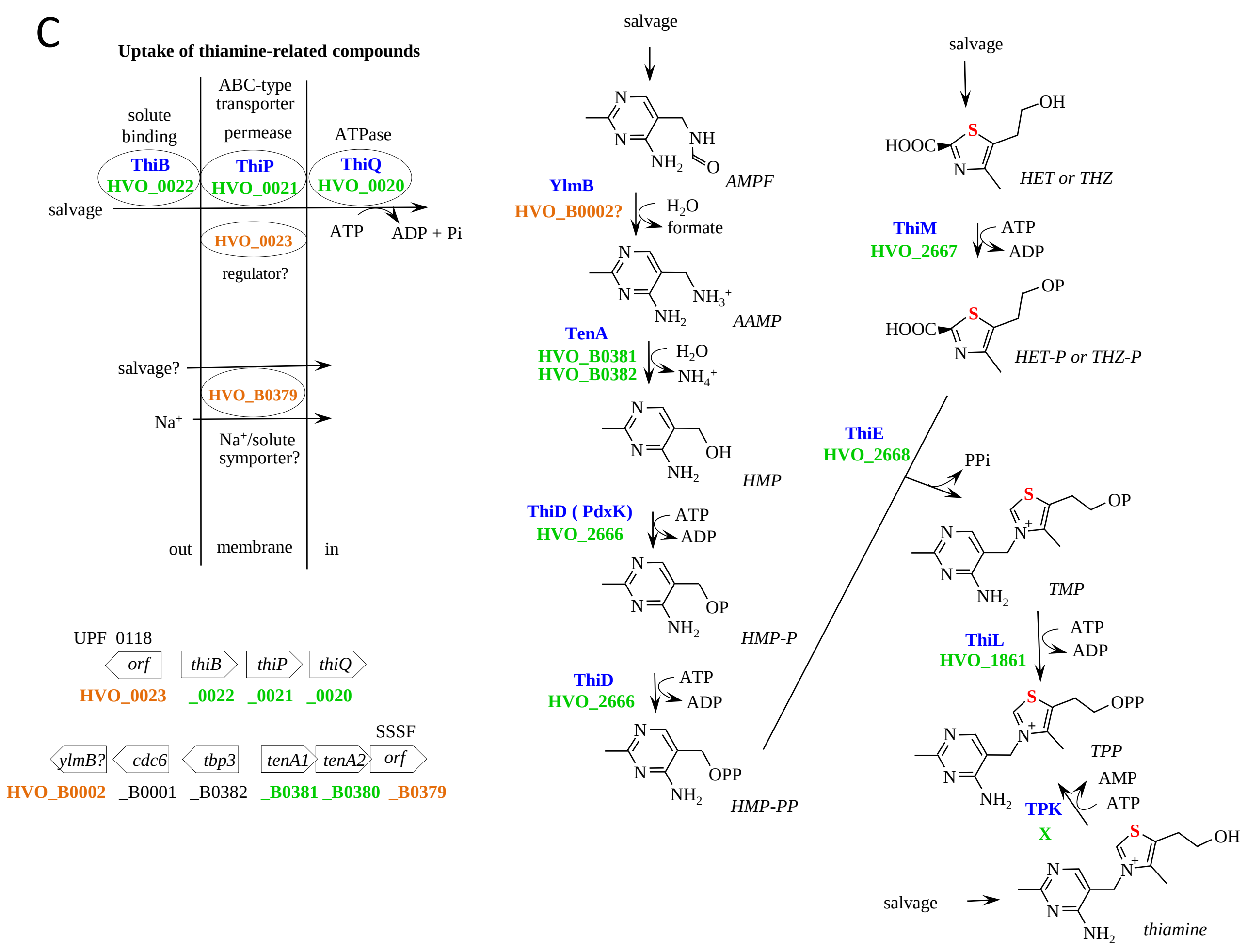


**Figure S1.** ***De novo*** **biosynthesis (A, B) and salvage (C) pathways of thiamine metabolism.** Thiamine biosynthetic enzymes are summarized in Table S1 with reference to function, gene locus tag and UnitProtKB accession numbers. Enzymes of bacteria and yeast are indicated in blue and purple, respectively. *Hfx. volcanii* ORF code homologs associated with thiamine metabolism based on homology are indicated in green (where X indicates no homolog detected and ? is used when the enzyme is yet unassigned), while those based on gene synteny and limited homology are indicated in orange. THI4-SH indicates the catalytic cysteine side chain. THI4-C=CH indicates the dehydroalanine form of the enzyme after sulfur transfer. The sulfur atom associated with formation of the thiazole ring is highlighted in red. Abbreviations: 4-amino-5-aminomethyl-2-methylpyrimidine (AAMP); ADP-thiazole (ADT); 5-amino-1-(5-phospho-D-ribosyl)imidazole (AIR, synonym 5-aminoimidazole ribotide); N-[(4-amino- 2-methylpyrimidin-5-yl)methyl]formamide (AMPF); deoxy-D-xylulose 5-phosphate (DXP); 4-amino-5-hydroxymethyl-2-methylpyrimidine (HMP); ); 4-amino-5-hydroxymethyl-2-methylpyrimidine phosphate (HMP-P); ); 4-amino-5-hydroxymethyl-2-methylpyrimidine pyrophosphate (HMP-PP); nicotinamide adenine dinucleotide (NAD); pyridoxal 5'-phosphate (PLP); S-adenosyl-methionine (SAM); 4-methyl-5-(β-hydroxyethyl)thiazole (THZ); 4-methyl-5-(β-hydroxyethyl)thiazole phosphate (THZ-P or HET-P); thiazole tautomer (R,Z)-2-(2-carboxy-4-methylthiazol-5(2H)-ylidene)ethyl phosphate (cTHZ*-P); thiamine monophosphate (TMP); thiamine pyrophosphate (TPP). *Hfx. volcanii* ORFs not listed in Table S1 include: HVO_0109 (D4GYV5, SufS-type cysteine desulfurase)[55]; HVO_B0001 (D4GP02, Orc1-type DNA replication protein); HVO_B0382 (D4GQ28, TATA-box-binding protein 3 or Tbp3). *Hfx. volcanii* homologs of bacterial glycine oxidase (ThiO), thiazole synthase (ThiG), thiamine phyrophosphokinase (TPK) and thiazole tautomerase (TenI) in addition to yeast HMP-P synthase (THI5) and thiamine pyrophosphokinase (THI80) were not detected. Conversion of ADT to THZ-P is predicted to be catalyzed by a NUDIX hydrolase domain enzyme that has yet to be identified [56]. Members of the RHD (IPR001763) and NUDIX hydrolase domain (IPR000086) are common in *Hfx. volcanii*. HVO_1651 is related to bacterial ThiI but devoid of the rhodanese-like domain (RHD), which alone mediates the catalytic function of ThiI in thiamine biosynthesis [10, 11].

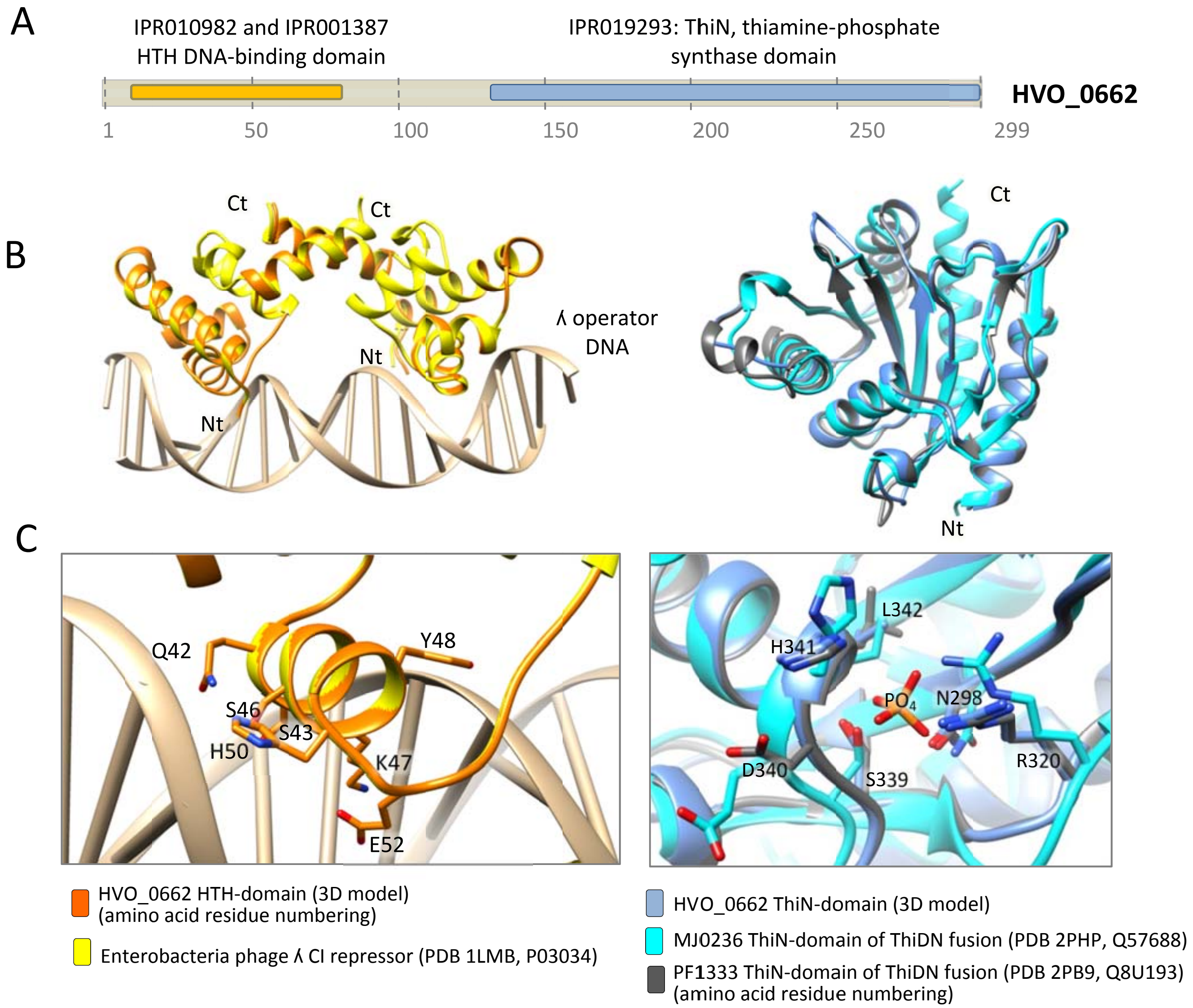


**Suppl. Figure S2**. Domain organization and 3D-structural modeling suggest *Hfx. volcanii* HVO_0662 functions as transcriptional regulator of thiamine metabolism. (A) HVO_0662 is organized as fusion of an N-terminal helix-turn-helix (HTH) DNA binding domain and C-terminal ThiN domain based on InterProScan domain recognition [4]. Protein homologs with this HTH-ThiN domain configuration are widespread in halophilic archaea and identified in species of Crenarchaeota (*Pyrobaculum*, *Thermofilum*, *Sulfolobus*, *Metallosphaera*, *Caldivirga*, *Hyperthermus*, *Vulcanisaeta* and *Acidianus*) and Euryarchaeota (*Thermococcus* and *Pyrococcus*). Other archaea and bacteria are predicted to encode ThiD-ThiN protein fusions. Overview (B) and selected close-up views (C) of the 3D structural models of HVO_0662 HTH (left) and ThiN (right) domains generated at 97.7% and >99.9% confidence, respectively, by Phyre2 based fold-recognition and model building [6]. Models were overlaid onto x-ray crystal structures with PDB, UniProtKB reference and amino acid residue numbering as indicated. The ThiN domain of the ThiDN fusion protein, TM0790, from the bacterium *Thermotoga maritima* catalyzes thiamine phosphate synthase (TPS) activity *in vitro* and complements an *E. coli ΔthiE* strain for thiamine auxotrophy (although additional factors in *E. coli* cell lysate as well as the N-terminal ThiD domain were important for full activity) [14]. The archaeal ThiDN (of *Pyrobaculum calidifontis*) is multifunctional in formation of TMP from HMP and THZ-P in the presence of Mg-ATP [15], and its ThiN domain is a functional analog of the bacterial ThiE, catalyzing formation of TMP with release of PPi from HMP-PP and THZ-P (also known as HET-P or 4-methyl-5-(β-hydroxyethyl)thiazole phosphate) [15]. X-ray crystal structure (PDB 2PB9) guided site-directed mutagenesis [15] suggest the *Pyrococcus* ThiN R320 and H341 are involved in the catalytic reaction (residues of structural analogy are predicted for HVO_0662; R183 and H204, respectively). Whether HTH-ThiN fusion proteins such as HVO_0662 synthesize TMP and/or bind intermediates/products of thiamine biosynthesis to modulate transcription remains to be determined.

## Supplementary References


1. Falb M, Müller K, Königsmaier L, Oberwinkler T, Horn P, von Gronau S, Gonzalez O, Pfeiffer F, Bornberg-Bauer E, Oesterhelt D: **Metabolism of halophilic archaea**. *Extremophiles* 2008, **12**(2):177-196.
2. Siddaramappa S, Challacombe JF, DeCastro RE, Pfeiffer F, Sastre DE, Gimenez MI, Paggi RA, Detter JC, Davenport KW, Goodwin LA *et al*: **A comparative genomics perspective on the genetic content of the alkaliphilic haloarchaeon *Natrialba magadii* ATCC 43099T**. *BMC Genomics* 2012, **13**:165.
3. Hartman A, Norais C, Badger J, Delmas S, Haldenby S, Madupu R, Robinson J, Khouri H, Ren Q, Lowe T *et al*: **The complete genome sequence of *Haloferax volcanii* DS2, a model archaeon**. *PLoS One* 2010, **5**(3):e9605.
4. Quevillon E, Silventoinen V, Pillai S, Harte N, Mulder N, Apweiler R, Lopez R: **InterProScan: protein domains identifier**. *Nucleic Acids Res* 2005, **33**(Web Server issue):W116-120.
5. Marchler-Bauer A, Lu S, Anderson JB, Chitsaz F, Derbyshire MK, DeWeese-Scott C, Fong JH, Geer LY, Geer RC, Gonzales NR *et al*: **CDD: a Conserved Domain Database for the functional annotation of proteins**. *Nucleic Acids Res* 2011, **39**(Database issue):D225-229.
6. Kelley LA, Sternberg MJ: **Protein structure prediction on the Web: a case study using the Phyre server**. *Nat Protoc* 2009, **4**(3):363-371.
7. Humbard M, Miranda H, Lim J, Krause D, Pritz J, Zhou G, Chen S, Wells L, Maupin-Furlow J: **Ubiquitin-like small archaeal modifier proteins (SAMPs) in *Haloferax volcanii***. *Nature* 2010, **463**(7277):54-60.
8. Miranda HV, Antelmann H, Hepowit N, Chavarria NE, Krause DJ, Pritz JR, Bäsell K, Becher D, Humbard MA, Brocchieri L *et al*: **Archaeal ubiquitin-like SAMP3 is isopeptide-linked to proteins via a UbaA-dependent mechanism**. *Mol Cell Proteomics* 2014, **13**(1):220-239.
9. Miranda H, Nembhard N, Su D, Hepowit N, Krause D, Pritz J, Phillips C, Söll D, Maupin-Furlow J: **E1- and ubiquitin-like proteins provide a direct link between protein conjugation and sulfur transfer in archaea.** *Proc Natl Acad Sci U S A* 2011, **108**(11):4417-4422.
10. Martinez-Gomez NC, Palmer LD, Vivas E, Roach PL, Downs DM: **The rhodanese domain of ThiI is both necessary and sufficient for synthesis of the thiazole moiety of thiamine in *Salmonella enterica***. *J Bacteriol* 2011, **193**(18):4582-4587.
11. Bender RA: **The danger of annotation by analogy: most ";*thi*I" genes play no role in thiamine biosynthesis**. *J Bacteriol* 2011, **193**(18):4574-4575.
12. Webb E, Claas K, Downs D: ***thiBPQ* encodes an ABC transporter required for transport of thiamine and thiamine pyrophosphate in *Salmonella typhimurium***. *J Biol Chem* 1998, **273**(15):8946-8950.
13. Xi J, Ge Y, Kinsland C, McLafferty FW, Begley TP: **Biosynthesis of the thiazole moiety of thiamin in *Escherichia coli*: identification of an acyldisulfide-linked protein--protein conjugate that is functionally analogous to the ubiquitin/E1 complex**. *Proc Natl Acad Sci U S A* 2001, **98**(15):8513-8518.
14. Morett E, Korbel JO, Rajan E, Saab-Rincon G, Olvera L, Olvera M, Schmidt S, Snel B, Bork P: **Systematic discovery of analogous enzymes in thiamin biosynthesis**. *Nat Biotechnol* 2003, **21**(7):790-795.
15. Hayashi M, Kobayashi K, Esaki H, Konno H, Akaji K, Tazuya K, Yamada K, Nakabayashi T, Nosaka K: **Enzymatic and structural characterization of an archaeal thiamin phosphate synthase**. *Biochim Biophys Acta* 2014, **1844**(4):803-809.

16. Chatterjee A, Abeydeera ND, Bale S, Pai PJ, Dorrestein PC, Russell DH, Ealick SE, Begley TP: ***Saccharomyces cerevisiae* THI4p is a suicide thiamine thiazole synthase**. *Nature* 2011, **478**(7370):542-546.
17. Bender RA: **The danger of annotation by analogy: most *thiI* genes play no role in thiamine biosynthesis**. *J Bacteriol* 2011, **193**(18):4574-4575.
18. Liu Y, Zhu X, Nakamura A, Orlando R, Söll D, Whitman WB: **Biosynthesis of 4-thiouridine in tRNA in the methanogenic archaeon *Methanococcus maripaludis***. *J Biol Chem* 2012, **287**(44):36683-36692.
19. Webb E, Downs D: **Characterization of *thiL*, encoding thiamin-monophosphate kinase, in *Salmonella typhimurium***. *J Biol Chem* 1997, **272**(25):15702-15707.
20. Chatterjee A, Li Y, Zhang Y, Grove TL, Lee M, Krebs C, Booker SJ, Begley TP, Ealick SE: **Reconstitution of ThiC in thiamine pyrimidine biosynthesis expands the radical SAM superfamily**. *Nat Chem Biol* 2008, **4**(12):758-765.
21. Lawhorn BG, Mehl RA, Begley TP: **Biosynthesis of the thiamin pyrimidine: the reconstitution of a remarkable rearrangement reaction**. *Org Biomol Chem* 2004, **2**(17):2538-2546.
22. Zhang Y, Begley TP: **Cloning, sequencing and regulation of *thiA*, a thiamin biosynthesis gene from *Bacillus subtilis***. *Gene* 1997, **198**(1-2):73-82.
23. Smith JM, Daum HA: **Nucleotide sequence of the purM gene encoding 5'-phosphoribosyl-5-aminoimidazole synthetase of *Escherichia coli* K12**. *J Biol Chem* 1986, **261**(23):10632-10636.
24. Schrimsher JL, Schendel FJ, Stubbe J, Smith JM: **Purification and characterization of aminoimidazole ribonucleotide synthetase from *Escherichia coli***. *Biochemistry* 1986, **25**(15):4366-4371.
25. Taylor SV, Kelleher NL, Kinsland C, Chiu HJ, Costello CA, Backstrom AD, McLafferty FW, Begley TP: **Thiamin biosynthesis in *Escherichia coli*. Identification of ThiS thiocarboxylate as the immediate sulfur donor in the thiazole formation**. *J Biol Chem* 1998, **273**(26):16555-16560.
26. Park JH, Dorrestein PC, Zhai H, Kinsland C, McLafferty FW, Begley TP: **Biosynthesis of the thiazole moiety of thiamin pyrophosphate (vitamin B1)**. *Biochemistry* 2003, **42**(42):12430-12438.
27. Humbard M, Miranda H, Lim J, Krause D, Pritz J, Zhou G, Chen S, Wells L, Maupin-Furlow J: **Ubiquitin-like small archaeal modifier proteins (SAMPs) in *Haloferax volcanii***. *Nature* 2010, **463**:54-60.
28. Jeong YJ, Jeong BC, Song HK: **Crystal structure of ubiquitin-like small archaeal modifier protein 1 (SAMP1) from *Haloferax volcanii***. *Biochem Biophys Res Commun* 2011, **405**(1):112-117.
29. Li Y, Maciejewski MW, Martin J, Jin K, Zhang Y, Maupin-Furlow JA, Hao B: **Crystal structure of the ubiquitin-like small archaeal modifier protein 2 from *Haloferax volcanii***. *Protein Sci* 2013, **22**(9):1206-1217.
30. Liao S, Zhang W, Fan K, Ye K, Zhang X, Zhang J, Xu C, Tu X: **Ionic strength-dependent conformations of a ubiquitin-like small archaeal modifier protein (SAMP2) from Haloferax volcanii**. *Sci Rep* 2013, **3**:2136.
31. Park JH, Burns K, Kinsland C, Begley TP: **Characterization of two kinases involved in thiamine pyrophosphate and pyridoxal phosphate biosynthesis in *Bacillus subtilis*: 4-amino-5-hydroxymethyl-2methylpyrimidine kinase and pyridoxal kinase**. *J Bacteriol* 2004, **186**(5):1571-1573.
32. Kawasaki Y, Onozuka M, Mizote T, Nosaka K: **Biosynthesis of hydroxymethylpyrimidine pyrophosphate in *Saccharomyces cerevisiae***. *Curr Genet* 2005, **47**(3):156-162.
33. Llorente B, Fairhead C, Dujon B: **Genetic redundancy and gene fusion in the genome of the Baker's yeast *Saccharomyces cerevisiae*: functional characterization of a three-member gene family involved in the thiamine biosynthetic pathway**. *Mol Microbiol* 1999, **32**(6):1140-1152.

34. Mizote T, Nakayama H: **The *thiM* locus and its relation to phosphorylation of hydroxyethylthiazole in *Escherichia coli***. *J Bacteriol* 1989, **171**(6):3228-3232.
35. Yazdani M, Zallot R, Tunc-Ozdemir M, de Crécy-Lagard V, Shintani DK, Hanson AD: **Identification of the thiamin salvage enzyme thiazole kinase in *Arabidopsis* and maize**. *Phytochemistry* 2013, **94**:68-73.
36. Backstrom AD, McMordie RAS, Begley TP: **Biosynthesis of thiamin I: the function of the *thiE* gene product**. *J Am Chem Soc* 1995, **117**:2351-2352.
37. Reddick JJ, Nicewonger R, Begley TP: **Mechanistic studies on thiamin phosphate synthase: evidence for a dissociative mechanism**. *Biochemistry* 2001, **40**(34):10095-10102.
38. Toms AV, Haas AL, Park JH, Begley TP, Ealick SE: **Structural characterization of the regulatory proteins TenA and TenI from *Bacillus subtilis* and identification of TenA as a thiaminase II**. *Biochemistry* 2005, **44**(7):2319-2329.
39. Jenkins AH, Schyns G, Potot S, Sun G, Begley TP: **A new thiamin salvage pathway**. *Nat Chem Biol* 2007, **3**(8):492-497.
40. Onozuka M, Konno H, Kawasaki Y, Akaji K, Nosaka K: **Involvement of thiaminase II encoded by the *THI20* gene in thiamin salvage of *Saccharomyces cerevisiae***. *FEMS Yeast Res* 2008, **8**(2):266-275.
41. Coquille S, Roux C, Fitzpatrick TB, Thore S: **The last piece in the vitamin B1 biosynthesis puzzle: structural and functional insight into yeast 4-amino-5-hydroxymethyl-2-methylpyrimidine phosphate (HMP-P) synthase**. *J Biol Chem* 2012, **287**(50):42333-42343.
42. Hazra AB, Han Y, Chatterjee A, Zhang Y, Lai RY, Ealick SE, Begley TP: **A missing enzyme in thiamin thiazole biosynthesis: identification of TenI as a thiazole tautomerase**. *J Am Chem Soc* 2011, **133**(24):9311-9319.
43. Settembre EC, Dorrestein PC, Park JH, Augustine AM, Begley TP, Ealick SE: **Structural and mechanistic studies on ThiO, a glycine oxidase essential for thiamin biosynthesis in *Bacillus subtilis***. *Biochemistry* 2003, **42**(10):2971-2981.
44. Nishiya Y, Imanaka T: **Purification and characterization of a novel glycine oxidase from *Bacillus subtilis***. *FEBS Lett* 1998, **438**(3):263-266.
45. Challand MR, Martins FT, Roach PL: **Catalytic activity of the anaerobic tyrosine lyase required for thiamine biosynthesis in *Escherichia coli***. *J Biol Chem* 2010, **285**(8):5240-5248.
46. Kriek M, Martins F, Challand MR, Croft A, Roach PL: **Thiamine biosynthesis in *Escherichia coli*: identification of the intermediate and by-product derived from tyrosine**. *Angew Chem Int Ed Engl* 2007, **46**(48):9223-9226.
47. Kawasaki Y: **Copurification of hydroxyethylthiazole kinase and thiamine-phosphate pyrophosphorylase of *Saccharomyces cerevisiae*: characterization of hydroxyethylthiazole kinase as a bifunctional enzyme in the thiamine biosynthetic pathway**. *J Bacteriol* 1993, **175**(16):5153-5158.
48. Nosaka K, Nishimura H, Kawasaki Y, Tsujihara T, Iwashima A: **Isolation and characterization of the THI6 gene encoding a bifunctional thiamin-phosphate pyrophosphorylase/hydroxyethylthiazole kinase from *Saccharomyces cerevisiae***. *J Biol Chem* 1994, **269**(48):30510-30516.
49. Nosaka K, Kaneko Y, Nishimura H, Iwashima A: **Isolation and characterization of a thiamin pyrophosphokinase gene, THI80, from *Saccharomyces cerevisiae***. *J Biol Chem* 1993, **268**(23):17440-17447.
50. Baker LJ, Dorocke JA, Harris RA, Timm DE: **The crystal structure of yeast thiamin pyrophosphokinase**. *Structure* 2001, **9**(6):539-546.
51. Müller IB, Bergmann B, Groves MR, Couto I, Amaral L, Begley TP, Walter RD, Wrenger C: **The vitamin B1 metabolism of *Staphylococcus aureus* is controlled at enzymatic and transcriptional levels**. *PLoS One* 2009, **4**(11):e7656.

52. Wolf YI, Makarova KS, Yutin N, Koonin EV: **Updated clusters of orthologous genes for Archaea: a complex ancestor of the Archaea and the byways of horizontal gene transfer**. *Biol Direct* 2012, **7**:46.
53. Finn MW, Tabita FR: **Synthesis of catalytically active form III ribulose 1,5-bisphosphate carboxylase/oxygenase in archaea**. *J Bacteriol* 2003, **185**(10):3049-3059.
54. Sato T, Atomi H, Imanaka T: **Archaeal type III RuBisCOs function in a pathway for AMP metabolism**. *Science* 2007, **315**(5814):1003-1006.
55. Zafrilla B, Martínez-Espinosa RM, Esclapez J, Pérez-Pomares F, Bonete MJ: **SufS protein from *Haloferax volcanii* involved in Fe-S cluster assembly in haloarchaea**. *Biochim Biophys Acta* 2010, **1804**(7):1476-1482.
56. Begley TP, Ealick SE, McLafferty FW: **Thiamin biosynthesis: still yielding fascinating biological chemistry**. *Biochem Soc Trans* 2012, **40**(3):555-560.
